# Advances in nucleic acid probe-based detection of gene point mutations: a review

**DOI:** 10.3389/fchem.2025.1672155

**Published:** 2025-09-25

**Authors:** Xuyang Pu, Xueqiang Wu

**Affiliations:** 1 Affiliated Meizhou Hospital of Shantou University Medical College, Shantou University, Meizhou, China; 2 Institute of Basic Medical Sciences, Meizhou People’s Hospital, Meizhou, China; 3 Guangdong Engineering Technological Research Center of Clinical Molecular Diagnosis and Antibody Drugs, Meizhou Academy of Medical Sciences, Meizhou, China; 4 Breast Center, MeiZhou People’s Hospital, Meizhou, China

**Keywords:** mutation detection, point mutations, low-level mutation, enzymes, nucleic acid analogs, nanomaterials

## Abstract

A fundamental characteristic of gene mutations is the permanent alteration of the DNA sequence, including point mutations, deletions, inversions, and translocations. Among these, DNA point mutation detection has consistently remained a central focus of research across multiple disciplines due to its close association with a range of diseases, such as sickle cell anemia and β-thalassemia. However, the typically low abundance of such mutations presents a significant technical challenge. Due to technical limitations in detection sensitivity, increasing research efforts have been directed toward nucleic acid probe-based strategies to enhance the efficiency and accuracy of point mutation identification. This review summarizes the developments in nucleic acid probe-based techniques for detecting gene point mutations, with an emphasis on strategies involving pure nucleic acid probes as well as the synergistic use of enzymes, nucleic acid analogs, and nanotechnology. The principles, advantages, and limitations of the above technologies are also described and summarized. In addition, we also explored the application of AI technology in nucleic acid probes and the potential future challenges.

## 1 Introduction

Point mutation refers to an alteration that occurs at a single nucleotide of a DNA molecule, characterized by the substitution, insertion, or deletion of only one nucleotide in the DNA sequence. Mutated genes typically trigger a cascade of detectable biological alterations, including changes in protein expression and function, changes in RNA expression, epigenetic modifications (e.g., DNA methylation, histone modifications), and variations in metabolite profiles (Eriksson et al., 2017; Ho et al., 2008). Numerous genetic disorders and cancers originate from gene mutations that cause dysregulation of functional proteins. Among the most frequently mutated genes, *EGFR*, *TP53*, and *KRAS* are associated with diverse malignancies including non-small cell lung cancer (NSCLC) (Bai et al., 2024; Mao et al., 2021), colorectal carcinoma (Adorisio et al., 2025; Kim et al., 2020), and pancreatic cancer (Hashimoto et al., 2016; McCubrey et al., 2022). Detection of point mutations facilitates the early diagnosis of genetic diseases and the screening of carriers, while also contributing to the molecular subtyping of tumor-related diseases and guiding precision medicine. In addition, resistance in bacteria, viruses, and parasites is often mediated by point mutations (Carter et al., 2015; Menard and Dondorp, 2017), and identifying these mutations is valuable for clinical treatment and epidemiological prevention and control. Nevertheless, this process confronts significant technical challenges, notably the low concentration level of the target and interference from complex sample matrices.

The introduction of Sanger sequencing in 1977 revolutionized the field of genetic testing (Sanger et al., 1977). Nevertheless, Sanger sequencing has many limitations, such as a time-consuming process and high cost, which make it difficult to meet the demands for large-scale testing. Next-Generation Sequencing (NGS) has dramatically increased throughput through massive parallel sequencing, but it also has some drawbacks, such as shorter read lengths and higher requirements for sample and data analysis. Third-Generation Sequencing (TGS) overcomes some bottlenecks of NGS due to its ultra-long read length and non-reliance on PCR amplification (Van Dijk et al., 2018). However, TGS is still in the stage of rapid development, and its detection cost and high error rate need to be further optimized.

**FIGURE 1:**
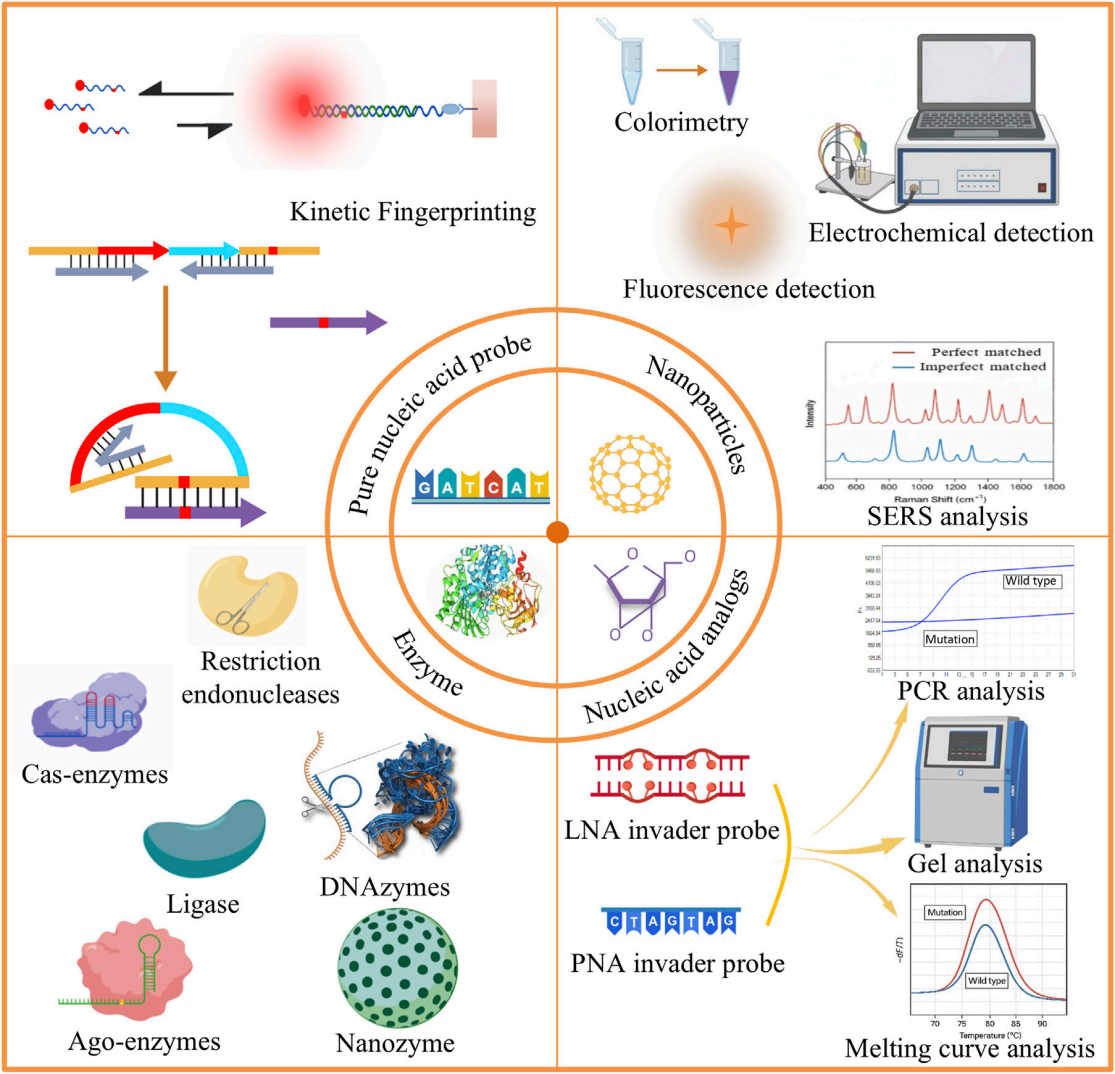
Schematic overview of different strategies for detecting gene point mutations: (1) Direct sequencing; (2) Nanoparticle-based methods; (3) Enzyme-based methods; (4) Nucleic acid analog-based methods.

In recent years, nucleic acid probe-based mutation detection technologies have made significant progress. Nucleic acid probes play a central role in distinguishing perfectly matched sequences from single-base mismatches. Some research teams have developed pure nucleic acid probes that distinguish mismatches based on kinetic differences. However, the thermodynamic and kinetic differences caused by a single nucleotide variation are often subtle, requiring additional strategies to improve specificity and sensitivity. From a mechanistic perspective, existing approaches can be broadly divided into three categories. The first is enzyme-assisted methods, which exploit the high selectivity of ligases and exonucleases toward base pairing states, thereby achieving mismatch discrimination and signal amplification through catalytic reactions. The second is nanoparticle-based methods, which take advantage of the unique optical, electrical, or surface properties of nanomaterials to enable signal transduction and amplification. The third is nucleic acid analogue-based methods, in which chemically modified probes such as peptide nucleic acids (PNA) are employed to modulate hybridization stability and enhance single-base discrimination at the molecular level (Figure 1).

In this review, we will start with the above technologies and systematically summarize the progress of nucleic acid probe-based point mutation detection methods, analyze the advantages and disadvantages of different technologies, as well as explore the potential directions for optimization and development.

## 2 Enzyme-mediated point mutation detection

Enzymes are widely available biocatalysts in biological systems, and their catalytic activity and high specificity make them valuable tools for gene mutation detection. Enzyme-based mutation detection methods typically require no complex instrumentation and are suitable for high-throughput and on-site detection. However, enzymes also present some inherent limitations. They are often highly dependent on specific sequences and may exhibit insufficient sensitivity when detecting rare mutations in complex samples. Nevertheless, with the development of enzyme engineering technology, enzyme-mediated nucleic acid probe-based mutation detection continues to hold significant promise.

### 2.1 Guided enzymes

**FIGURE 2:**
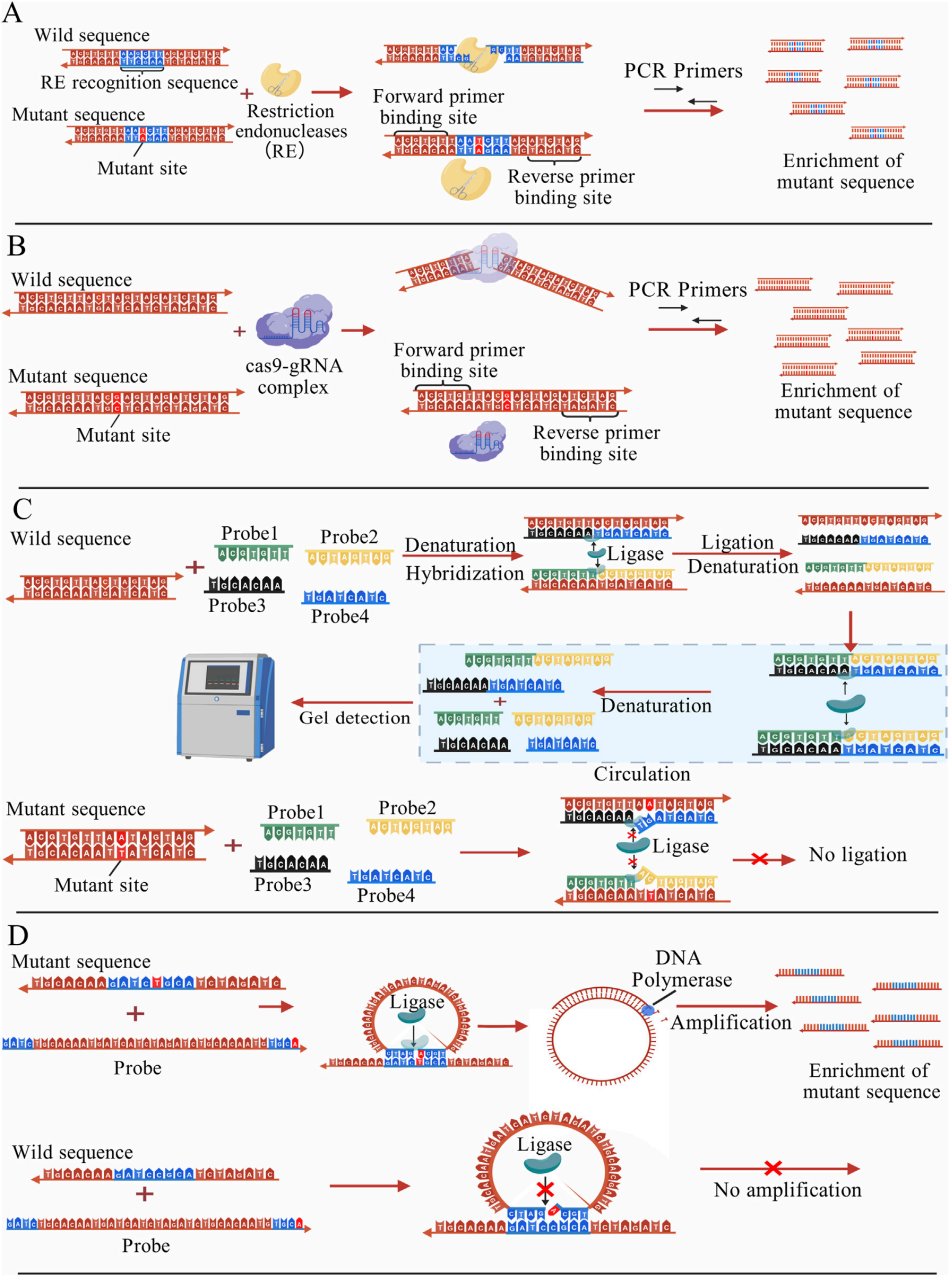
**(A)** Nucleic acid fragments with specific sequences are cleaved by REs and cannot be amplified by PCR, whereas fragments carrying mutation sites are not cleaved and can be selectively amplified and enriched for subsequent detection (Evans et al., 2018); **(B)** Under the guidance of sgRNA, the Cas9 protein cleaves the wild-type sequence, while the mutant sequence remains intact and can undergo normal PCR amplification, enrichment, and subsequent detection (Bae et al., 2019); **(C)** Two pairs of probes can only hybridize with perfectly matched sequences, and under the action of ligase, they are joined to form two new complementary nucleic acid strands. Through cyclic reactions, more probe pairs are ligated into new strands, which can then be analyzed by gel detection. When a point mutation is present, the probes cannot pair correctly, leading to ligation failure (Ruiz et al., 2020); **(D)** The probe perfectly matches the mutant sequence and is ligated into a new circular nucleic acid, which is then subjected to RCA amplification. The probe cannot fully match the wild-type sequence, resulting in ligation failure. (Ma and Gao, 2018); The figures are created with BioGDP.com (Jiang et al., 2025).

Restriction endonucleases (REs) typically enable mutation identification and screening by directly recognizing specific nucleic acid sequences and cleaving wild-type sequences. The sequence with point mutations cannot be cleaved by RE, it can be normally amplified and enriched by PCR for subsequent detection (Evans et al., 2018), as shown in Figure 2A. However, the conventional RE-based methods suffer from limited sensitivity and require complex designs to enable the detection of low-abundance mutations (Smith et al., 2020). RNA-guided nucleases are a class of enzymes that can bind to specific RNA molecules, recognize and act on target nucleic acids. The principle is that specifically designed RNA probe molecules serve as guides to precisely direct enzymes to recognize and cleave or degrade target DNA or RNA sequences. The most representative of these enzymes are Cas enzymes in the CRISPR-Cas system and the Argonaute (Ago) enzymes in the RNA interference system.

#### 2.1.1 Cas-enzymes-based detection

Compared with conventional methods, single-stranded guide RNAs (sgRNAs) based on PAM sequences can significantly improve the editing efficiency of mutant alleles, with a relative specificity capable of reaching 15-fold (Bae et al., 2019). One such method is DASH (depletion of abundant sequences by hybridization), which increases detection sensitivity by designing specific sgRNAs and mediating Cas9 protein cleavage. Guided by the sgRNA, the Cas protein cleaves and removes the unwanted wild-type sequences, thereby allowing the mutant sequences to be preserved and subsequently amplified by PCR for detection (Figure 2B). Because the mutation site is altered to cause loss of the PAM site (NGG), Cas9 cannot cleave sequences containing this mutation, which enables the removal of wild sequences (Gu et al., 2016). The CRISPR/Cas9 system can also be used in conjunction with technologies such as Amplified Fragment Length Polymorphism Analysis (AFLPA) and Sanger sequencing for the enrichment of mutant sequences. When used in conjunction with PCR, it can achieve a sensitivity of 0.1% (Jia et al., 2018).

Although CRISPR/Cas9 is a powerful tool, it has many shortcomings such as operational complexity and the limitation of relying on specific PAM sites. The use of Cas9 variants with relaxed PAM sequence requirements, such as xCas9, Cas9-NG can effectively address this issue, but at the cost of reduced DNA targeting and cleavage capabilities (Legut et al., 2020). In addition, off-target effects are one of the most urgent problems to be solved using CRISPR/Cas9 (Guo et al., 2023; Kim et al., 2015; Kimberland et al., 2018). Even for highly specific sgRNAs, Cas9 can generate cuts in DNA sequences similar to the target sequence, triggering unwanted gene mutations or expression. Some software has been developed to predict the off-target effects of CRISPR/Cas9 using machine learning methods, but the accuracy of the assay still needs to be improved (Fan and Xu, 2021).

#### 2.1.2 Ago-enzymes-based detection

The Ago family is a group of proteins widely involved in the regulation of gene expression, especially in RNA interference and microRNA-mediated gene silencing. The Ago proteins inhibit the expression of specific genes by binding to small RNA molecules, and subsequently recognizing and cleaving target mRNA. Different Ago family members can be categorized into different types according to their function. Such as PfAgo, TtAgo, and HsAgo2. Ago proteins from different species have different thermal stabilities, catalytic activities, and gene-editing potentials.

The Pyrococcus furiosus Argonaute (PfAgo) protein was used to propose a novel detection technique called PfAgo-mediated Nucleic Acid Detection (PAND) (He et al., 2019). The PfAgo protein can specifically recognize and cleave target DNA under the guidance of nucleic acid molecules, simultaneously generating short single-stranded DNA (ssDNA). The produced ssDNA is then able to bind to the corresponding fluorescent probes and guide the PfAgo protein for a second round of cleavage. The presence of the target DNA can be confirmed by detecting changes in fluorescence. Notably, by designing multiple pairs of guide sequences and fluorescent probes simultaneously, the PAND system enables simultaneous detection of multiple mutations in a single experiment. Experimental results demonstrate that PAND can successfully detect single nucleotide polymorphisms (SNPs) in the *BRCA1* gene, as well as *KRAS* G12D and *EGFR* T790M mutations, with a low background noise level of 0.1%.

Ago proteins can also be integrated with various materials and molecules to enhance their functionality. A current example is the graphene field effect transistor (GFET) platform mediated by Ago (Kong et al., 2024). Ago proteins provided nanoscale binding channels, allowing the organization and directed formation of DNA probes that rapidly recognize target mutations. GFET is electrochemical sensor that allows real-time monitoring of electrical changes resulting from binding of DNA probe to target sequences. An array comprising multiple independent Ago-GFET sensing channels can be integrated on a single microchip via photolithography, enabling simultaneous modular and multiplex detection of tumor-associated miRNAs, circulating tumor DNA, and viral RNA from a large number of biological samples. Furthermore, by leveraging the cleavage and guiding properties of TtAgo, NAVIGATER (Nucleic Acid enrichment Via DNA Guided Argonaute from Thermus thermophilus) was demonstrated by Song et al. (2020). First, an excess of short DNA guide strands fully complementary to the wild-type sequence are designed and pre-bound with TtAgo to form a complex. This complex precisely cleaves perfectly matched wild-type DNA and RNA, while Mutant nucleic acid sequences remain intact. Following downstream Xenonucleic Acid PCR (XNA-PCR), NAVIGATOR enables ultra-sensitive detection of mutations as low as 0.01% in pancreatic cancer blood samples. More importantly, the system allows for the design of multiple DNA guide strands targeting different mutation sites, enabling simultaneous detection of multiple mutations in a single reaction system.

#### 2.1.3 Limitations and optimization of guided enzymes

Although guided enzymes have been widely applied in gene detection and gene editing, there are still many limitations. One major challenge lies in improving delivery efficiency (Finn et al., 2018) and it is hoped that new viral vectors or non-viral delivery systems can be developed in the future to optimize this process (Sun et al., 2025; Tabebordbar et al., 2016). Moreover, the presence of pre-existing antibodies and T-cell immunity against bacterially derived Cas proteins in humans poses the risk of undesired immune responses to therapeutic vectors (Shen et al., 2022). With the continued maturation of protein engineering strategies, these immunological barriers may be progressively mitigated, and potentially even eliminated (Crudele and Chamberlain, 2018).

### 2.2 Ligase

#### 2.2.1 Ligase chain reaction (LCR)

As a classical nucleic acid amplification technique, ligase chain reaction (LCR) has been used for a variety of gene mutation detection methods (Ruiz et al., 2020). The core principle is to design two pairs of oligonucleotide probes, each pair of probes targeting two complementary strands of mutated DNA. When the thermally denatured ssDNA hybridizes correctly with the probes, the ligase catalyzed the formation of phosphodiester bonds adjacent to the probes, and the two pairs of probes were ligated into two complete DNA strands. The number of intact probe strands increased exponentially through subsequent amplification cycles. Conversely, the probes do not completely hybridize with wild-type DNA sequences. The principle of LCR technology is illustrated in the schematic diagram in Figure 2C. In practical experiments, LCR is typically used in conjunction with other signal readout techniques (Liu Z.-J. et al., 2020).

#### 2.2.2 Combining LCR with fluorescence

The combination of ligases and fluorescence is a promising strategy for detecting nucleic acid mutations. Sawamura et al. reported a method based on ligase-mediated branching hybridization chain reaction (LDR) combined with fluorescence quenching technology for the rapid detection of point mutations in mixed samples. This method detects mutations through the use of both differentiated and universal primers, enables specific detection within PCR amplification products, facilitates the formation of MBs via primer ligation reactions, and achieves fluorescence quenching via Förster Resonance Energy Transfer (FRET). Experimental results have demonstrated that this approach is capable of detecting mutant DNA at levels as low as 5% (Sawamura and Hashimoto, 2017). In order to increase the detection limit, a branching hybridisation chain reaction has been applied to ligase mutation detection, referred to as the ligation-mediated branching hybridisation chain reaction (ligation-bHCR). This method combines the ligase discriminatory ability and the signal amplification function of bHCR that is capable of resolving single-nucleotide variants in mRNAs at single-molecule resolution (Tang et al., 2017).

#### 2.2.3 Combining ligase with RCA

Rolling Circle Amplification (RCA) is a common nucleic acid amplification technique that generates large numbers of DNA copies using circular DNA templates and enzyme-driven amplification reactions. The combination of RCA and ligase has been widely used for point mutation detection (Ma and Gao, 2018; Wu et al., 2010; Zou et al., 2014). This approach relies on the use of specially designed primer probes to guide the amplification of a circular DNA template. When the target DNA contains a mutation, the probe perfectly matches it, and the ligase catalyzes the formation of a phosphodiester bond. The probe is ligated into a new circular nucleic acid, which serves as a template for subsequent RCA amplification. In contrast, the probe cannot fully match the wild-type sequence and therefore cannot be efficiently ligated. The principle of DNA ligase-assisted RCA is shown in Figure 2D. Furthermore, RCA can also be combined with other molecules, such as nucleases and nanoparticles, to further enhance the detection sensitivity (Li et al., 2010; Shi et al., 2022).

As important tools in nucleic acid research, both LCR and RCA rely heavily on the catalytic function of ligase. It has been shown that the sensitivity of the assay can be further improved by combining the two research methods (Cheng et al., 2013). However, the complexity of ligase-based experimental procedures demands significant technical skills.

#### 2.2.4 Limitations and optimization of ligases

Theoretically, DNA ligases catalyze ligation events exclusively in the presence of perfect base pairing. However, the complexity of ligase-based experimental procedures demands significant technical skills (Wang and Zhang, 2014; Xu et al., 2016; Zhang et al., 2015). Furthermore, the most commonly used T4 DNA ligase is unstable at high temperatures and generally exhibits lower catalytic efficiency than DNA polymerases, resulting in an overall slower reaction rate compared with PCR (Lohman et al., 2014). These limitations may be addressed through advances in protein engineering. In addition, the continued exploration of thermophilic and extremophilic microorganisms is expected to facilitate the discovery and development of novel ligases (Tanabe et al., 2015).

### 2.3 DNAzyme

To overcome the limitations of traditional enzymes, such as poor stability, stringent storage requirements, and strict experimental conditions, increasing numbers of research groups have been dedicated to finding alternatives to conventional enzymes in recent years. DNAzymes represent a promising option. Unlike traditional protein enzymes, DNAzymes are obtained through *in vitro* selection methods (SELEX) and are composed of ssDNA molecules. Compared with natural protein enzymes, DNAzymes exhibit greater tolerance and stability (Li et al., 2025). They have already been widely applied in point mutation detection, where rational design allows them to be activated only under fully complementary conditions, thereby enabling highly specific recognition of single-base mismatches (Song et al., 2012).

#### 2.3.1 DNAzymes-based detection

DNAzyme-based mutation detection is generally integrated with other techniques or molecular components (Zhang et al., 2021), rather than relying solely on the DNAzyme itself, in order to achieve enhanced sensitivity and reliability in experimental results (Wang H.-Q. et al., 2011). To date, most methods developed involve complex probe labeling. Li et al. introduced a G-quadruplex DNAzyme into the Gap-LCR technique to achieve a detection strategy without chemically modified probes (Zhou et al., 2013). After Gap-LCR amplification, the DNAzyme exhibited peroxidase-like catalytic activity in the presence of hemin, catalyzing substrates (ABTS, tyramine) to produce colorimetric or fluorescent signals, thereby converting the presence of mutant DNA into visible or measurable outputs. Experimental results showed that this method was able to detect mutations in the presence of a 10^5^-fold excess of wild-type DNA.

Although detection based on G-quadruplex DNAzymes has many advantages over traditional enzymatic methods, the low catalytic activity of DNAzymes also limits their widespread application. Therefore, how to amplify DNAzymes has become a hotspot in analytical research. Xu et al. chose to amplify DNAzymes and enhance signals via target-catalyzed hairpin assembly (CHA), developing an enzyme-free and label-free DNAzyme sensor for SNP genotyping. This sensor successfully detected A-G single-nucleotide mutations on human chromosomes associated with Alzheimer’s disease in real saliva samples (Xu et al., 2017). Similarly, Li et al. adopted a comparable strategy, using flap endonuclease 1 (FEN1)-invader-triggered CHA to induce DNAzyme amplification, and developed a fluorescence amplification strategy based on multi-DNAzyme-linked structures. This method was successfully applied to the detection of point mutations in the *KARS* gene, achieving a detection limit as low as 4.23 fM (Li et al., 2022). Moreover, it performed excellently in complex serum samples, providing a new approach for isothermal and highly sensitive SNP detection.

#### 2.3.2 Limitations and future optimization of DNAzymes

Although DNAzymes possess multiple advantages compared with traditional enzymes, their catalytic activity still requires improvement (Wang et al., 2021). Most DNAzymes depend on specific metal ions (such as Mg^2+^ or Pb^2+^) as cofactors, and their activity may be reduced in the ionic environments of complex biological samples (Rosenbach et al., 2020). In addition, DNAzyme kinetic simulation experiments still show certain limitations in detecting DNA point mutations (Kenward and Dorfman, 2009). In the future, high-throughput SELEX and machine learning-assisted optimization of selection processes are expected to yield more efficient and specific DNAzymes. Similarly, DNAzymes are anticipated to be combined with tools such as MB and CRISPR-Cas systems to construct new composite detection platforms.

### 2.4 Nanozyme

Nanozymes refer to nanomaterials that possess catalytic activities similar to those of natural enzymes. Unlike natural enzymes or artificially recombined enzymes, nanozymes are nanoparticles or nanostructures with unique surface architectures and catalytic sites (Liang and Yan, 2019), but they can mimic the functions of natural enzymes such as peroxidases and oxidases (Hamed et al., 2024; Wang X. et al., 2023). Nanozymes exhibit several advantages, including resistance to inactivation, high thermal stability, protease tolerance, and low cost, making them excellent alternatives to traditional enzymes (Zandieh and Liu, 2024).

#### 2.4.1 Nanozyme-based detection

Through π-π stacking interactions, Guo et al. synthesized hemin-graphene hybrid nanosheets (H-GNs). Due to the presence of hemin, the H-GNs possess intrinsic peroxidase-like activity. In addition, H-GNs exhibit distinct affinities toward ssDNA and dsDNA. Based on these properties, the researchers developed a label-free colorimetric detection system for identifying single-base mutations in related DNA. Probe DNA adsorbs onto the H-GNs in single-stranded form and detaches only in the presence of fully complementary target DNA. Utilizing the catalytic activity of H-GNs, the TMB-H_2_O_2_ reaction is catalyzed to produce a color change indicating the presence or absence of a mutation. This detection system has been successfully applied for SNP detection in the HBV virus gene (Guo et al., 2011). Nanozymes composed of mesoporous silica and platinum nanoparticles (MS-PtNPs) synthesized by Chen et al. also exhibit peroxidase-like activity, which can be controlled by loading ssDNA. The adsorption of excessive DNA probes on MS blocks the nucleation sites of PtNP. Resulting in decreased peroxidase activity. Activity was restored when complementary target DNA was introduced. They combined it with a 3,3,5,5-tetramethylbenzidine/H2O2 color system to develop a rapid method for colorimetric determination of DNA. The method can detect relevant DNA at a minimum of 2.6 nM and can distinguish single base mismatches (Chen et al., 2018). Some research teams have constructed novel nanozymes by integrating existing enzymes onto a nanoscale platform (Petree et al., 2018). This approach enables the nanozymes to exhibit multiple enzymatic activities, which holds promise for broader and more diverse applications in future genetic detection.

#### 2.4.2 Limitations and optimization of nanozyme

Nanozymes, as exogenous nanomaterials, may contain heavy metals such as cadmium and mercury. Their metabolic processes within the body are not yet fully understood, and they may potentially trigger immune responses or cause organ accumulation and toxicity (Li et al., 2020). It is hoped that nanozymes composed of biodegradable materials, such as certain metal-organic frameworks (MOFs) or natural polymers, can be developed in the future (Liu Y. et al., 2020; Liu Y. et al., 2023). Additionally, the catalytic activity of nanozymes is highly dependent on their size, surface chemistry, and exposed surface area. It is extremely challenging to mass-produce nanozymes with entirely consistent structures in a highly reproducible manner. Developing synthesis methods that are precise, reproducible, and capable of ensuring batch-to-batch consistency represents a critical direction for future nanozyme optimization (Wang H. et al., 2024).

## 3 Nanomaterials-based point mutation detection

Gene mutation detection techniques have become increasingly diverse in recent years. Nanoparticles have attracted significant attention owing to their unique physicochemical properties, such as distinct optical behavior, surface modifiability, and superior electrochemical characteristics. The advent of nanomaterials has created new opportunities for signal transduction and amplification in gene mutation detection. The advantages and limitations of common nanoparticles are shown in Table 1. Their signal outputs are usually combined with different methods, such as colorimetry, electrochemistry, and surface-enhanced Raman scattering (SERS). In this subsection, nanoparticle-assisted nucleic acid probe-based mutation detection methods are introduced according to the different signal output strategies employed.

**TABLE 1:** Advantages and limitations of common nanoparticles^a^.

Nanoparticles	Advantages	Limitations	References
AuNP	Excellent biocompatibility, Photothermal properties, High stability, Strong surface modifiability, Surface plasmon resonance effect, Excellent optical and electrical properties	High-cost, undetermined long-term toxicity	Chen et al. (2023)
AgNP	Strong antibacterial activity, Excellent electrical conductivity, High catalytic activity, Surface-enhanced Raman scattering, Facile synthesis	Prone to oxidation and aggregation, Poor stability	Dikmen (2023), Martin et al. (2014)
CuNP	High catalytic activity, Excellent electrical and thermal conductivity, Low-cost, Excellent antibacterial activity, Abundant resource availability	Prone to oxidation, Poor stability, Prone to aggregation	Jessop et al. (2021)
MNP	Super paramagnetism, Excellent biocompatibility, High specific surface area and multifunctionality	Prone to oxidation, Prone to agglomeration, Poor hydrophilicity	Hwang et al. (2025)
GO	High mechanical strength, Multiple functionalization sites, Excellent water solubility, Ultrahigh specific surface area	Low electrical conductivity, Potential biotoxicity, Poor dispersibility	Pitiphattharabun et al. (2024)
QD	Size-tunable optical properties, High spectral purity, Excellent optical stability, High efficiency and low energy consumption, High quantum yield	High-cost, Complex synthesis processes	Phafat and Bhattacharya (2023)
SiO_2_NP	High photostability, Tunable size and morphology, Facile surface functionalization, Low biotoxicity, Good optical properties	low mechanical strength, Prone to agglomeration	Viana et al. (2024)
PtNP	High catalytic activity, Wide range of applications, Strong antioxidant properties	High-cost, Potential toxicity	Gu et al. (2019)
UCNP	Tunable spectral properties, High photostability, Superior imaging performance, Upconversion capability	High-cost, Complex synthesis processes	Liu et al. (2022)

^a^ Abbreviation: AuNP, gold nanoparticles; AgNP, silver nanoparticles; CuNP, copper nanoparticles; MNP, magnetic nanoparticles; GO, graphene oxide; QD, quantum dots; SiO_2_NP, silicon dioxide nanoparticles; PtNP, platinum nanoparticles; UCNP, upconversion nanoparticles.

### 3.1 Colorimetry

Colorimetry is an analytical method based on the visible color change of a substance after its reaction with a reagent. It is widely used for substance detection and clinical diagnosis because of its simplicity and cost-effectiveness. However, colorimetric methods often suffer from background interference and low sensitivity when detecting targets at low concentrations. Nanomaterials can significantly enhance the efficiency of colorimetric assays because of their unique physicochemical properties.

#### 3.1.1 AuNP-based colorimetry

**FIGURE 3:**
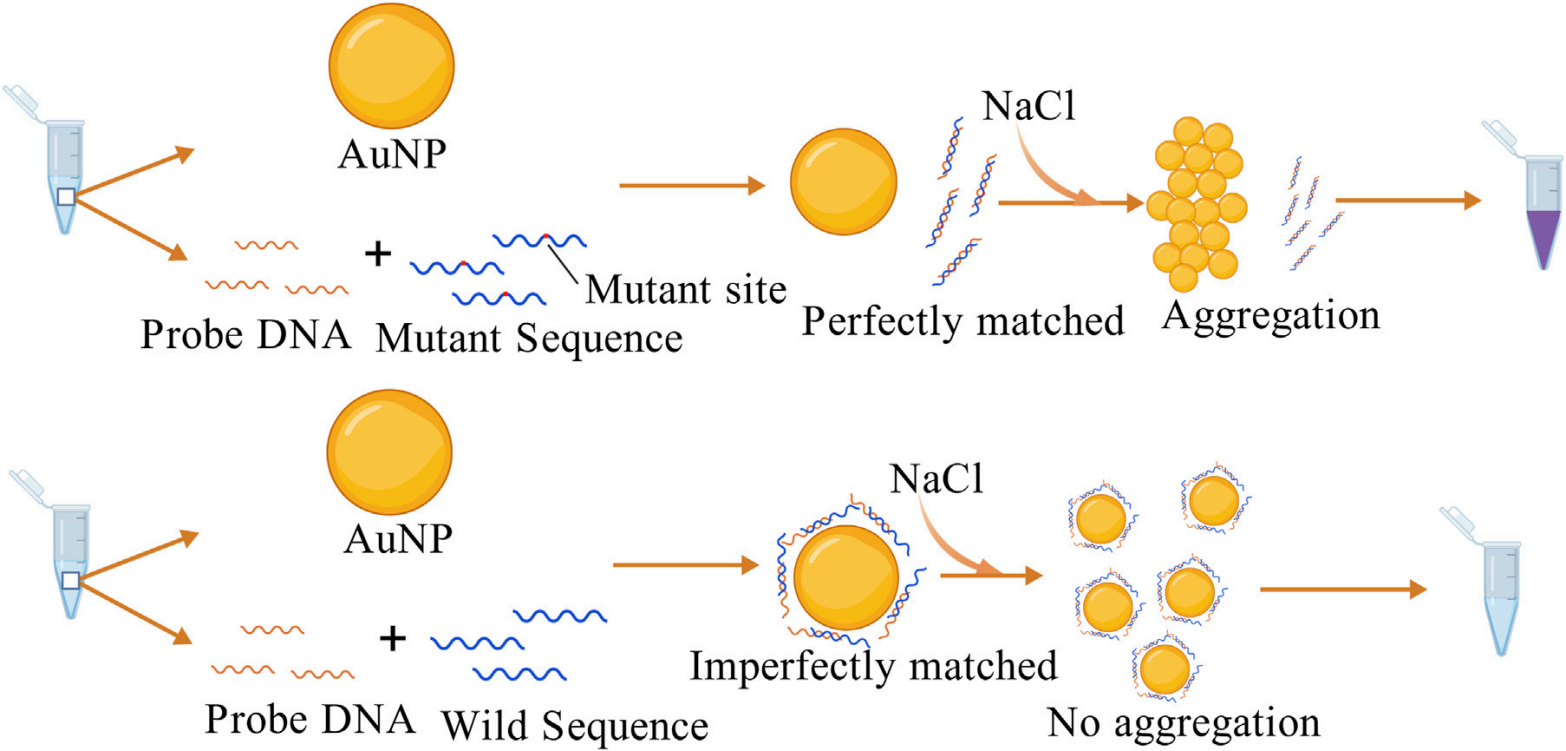
The probe perfectly matches the mutant sequence and cannot adsorb onto the AuNP surface, causing the AuNPs to lose their protective layer and aggregate with a color change in the presence of NaCl. In contrast, the probe partially complements the wild-type sequence, leaving some ssDNA able to adsorb onto the AuNP surface, inhibiting AuNP aggregation (Ramanathan et al., 2019). The figure is created with BioGDP.com.

In colorimetric assays based on nanoparticles, AuNPs are preferred because of their excellent biocompatibility, strong surface modification ability and high stability compared with other particles. As early as 1997, a study was conducted using AuNPs to differentiate target single-stranded oligonucleotides by colorimetric assay and obtained good detection efficiency (Elghanian et al., 1997). The changes in the aggregation behavior of DNA-modified AuNPs induced by salt allowed the distinction between fully complementary and terminal single-base mismatched double-stranded DNA (dsDNA). When the designed probe perfectly matches the target sequence, AuNPs aggregate and produce a color change (Sato, 2005). Since then, colorimetric methods have been increasingly used to detect small nucleic acid molecules and proteins. One example is the detection of *EGFR* mutations, which can be applied to the early diagnosis of NSCLC (Ramanathan et al., 2019). This study designed probe sequences complementary to the mutated sequences, forming a rigid double-stranded structure. In the presence of an appropriate concentration of NaCl, the dsDNA could not adsorb onto the surface of the AuNPs. Under high salt conditions, the AuNPs lost their protective layer and aggregated, causing a color change to purple. This successfully indicated the presence of *EGFR* mutations. The double-stranded structure formed between the wild-type sequence and the probe was unstable, and part of the ssDNA could still adsorb onto the surface of the AuNPs, which inhibited aggregation, as shown in Figure 3. Similar methods have also been applied to detect SNPs and other mutation sites in various diseases, such as psoriasis, breast cancer (Bai et al., 2020; Oh and Lee, 2011), and *KRAS* mutations (Valentini et al., 2013).

#### 3.1.2 Combining AuNP with other components-based colorimetry

Because of the excellent biocompatibility of AuNPs, researchers often combine them with other reaction components to construct systems with better performance (Zou et al., 2015). TEMPO-cellulose nanocrystals (TC-CNC)-based stabilized AuNPs probe (TC-AuNPs) was developed by Ganguly et al. The nanoparticles were induced to aggregate using electrostatic interactions between the pathogen DNA and the probe, with a sensitivity capable of reaching 20 fM (Ganguly et al., 2021). Furthermore, Cho and his colleagues explored a molecular mechanism-guided strategy by utilizing the mismatch recognition protein Mutator S (MutS) to specifically identify mismatch sites within double strands on the surface of AuNPs. Different mutation types (e.g., GT, CT, and CC mismatches) were quantitatively analyzed based on the melting temperature (T_m_) differences of nanoparticle assemblies. The T_m_ shift reached 14.7 °C, demonstrating the feasibility of protein-nanoparticle complexes in mutation-specific recognition (Cho et al., 2008).

#### 3.1.3 Limitations and future optimization of colorimetry

Though AuNPs provide excellent detection sensitivity for optical colorimetric analysis, however, there are also many limitations (Zhang et al., 2020). Detection signals are more prone to interference in sequences with high GC content or complex secondary structures (Chou et al., 2019). Moreover, most colorimetric assays merely provide binary (“yes/no”) or semi-quantitative strong/weak signals, making precise quantification challenging. Future strategies may involve integrating nucleic acid amplification techniques or CRISPR/Cas systems with colorimetric readouts to improve single-base resolution. Additionally, the selection of salts is regarded as one of the critical factors influencing reaction conditions (Yu et al., 2009). Unlike conventionally used sodium chloride, the application of sodium fluoride has increased sensitivity by 2.3-fold. This enhancement is attributed to the weak adsorption of fluoride ions on gold nanoparticles. Notably, polyadenine sequences provide stronger protection for gold nanoparticles than poly-thymine sequences, suggesting the potential value of sequence design in sensor optimization (Hu et al., 2020).

### 3.2 Fluorescence methods

Compared to traditional fluorescent dyes, nanoparticles (e.g., QDs, metal nanoparticles) offer higher fluorescence quantum yields and lower background noise, making them highly sensitive for detecting low-concentration mutations. QDs stand out for their high photostability and surface tunability, which allow them to bind to specific DNA probes and detect mutations by changes in fluorescence signals (Hosseini et al., 2017; Kim and Gang, 2018).

#### 3.2.1 QD-based fluorescence detection

**FIGURE 4:**
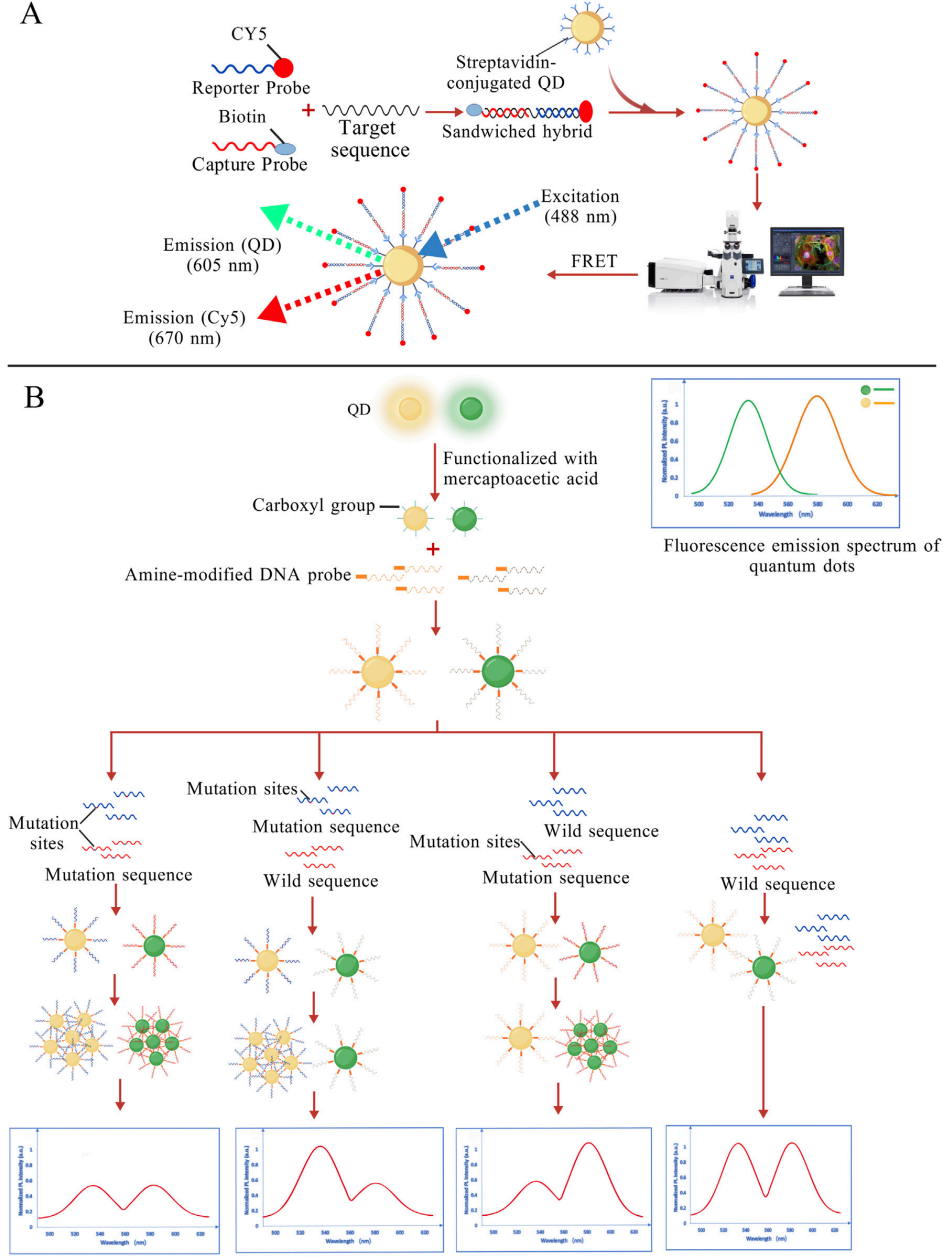
**(A)** The reporter probe and capture probe sandwich the target DNA and are conjugated to QDs. Under 488 nm laser excitation, signals from the QDs and Cy5 can be detected (Zhang R.-Y. et al., 2005); **(B)** Probes designed for different mutation sites are conjugated to distinct QDs. When the mutation sequence is present, the corresponding QDs aggregate, leading to fluorescence quenching, thereby enabling mutation detection. (Kang et al., 2012). The figures are created with BioGDP.com.

In 2005, Zhang et al. proposed a DNA nanosensor based on single QD and FRET. When the target DNA is present, the biotin-labeled capture probe and the Cy5-labeled reporter probe “sandwich” the target. The resulting complex binds to QDs modified with streptavidin on the surface, triggering a FRET effect. Under excitation with a 488 nm laser, both QD and Cy5 emissions can be detected. In the absence of target DNA, only the QD signal is observed. And the sensitivity of the single molecule detection is less than 50 copies, as shown in Figure 4A, which is a hundred-fold increase in sensitivity over standard MB (Zhang C.-Y. et al., 2005). Subsequent research teams developed QDs of different colors to label probes specific to mutant and wild-type alleles independently, enabling fluorescent signals to distinguish DNA types (Yeh, 2006). In a dispersed state, QDs generally have narrow bandwidth, high color purity in fluorescence spectra and large fluorescence quantum yield. When QDs aggregate, their fluorescence can be reduced to the point of becoming completely quenched. The mechanisms and factors contributing to fluorescence quenching may include FRET, Dexter energy transfer, QD concentration, and external environmental conditions (Kim et al., 2010; Liang et al., 2016; Zhou et al., 2022). Although fluorescence quenching is generally considered an unfavorable factor, in certain cases, it can be leveraged to develop highly sensitive detection methods. Utilizing the fluorescence quenching phenomenon of QDs aggregation, an assay has been proposed to simultaneously detect multiple mutations in the *EGFR* gene. The technique utilizes the spectrally resolved properties of QDs of different sizes to couple with specific molecular beacon probes targeting common *EGFR* mutations (L858R). The probes are designed for different mutation sites, and they are conjugated to distinct QDs. In the absence of mutation sequences, the QDs remain dispersed and continuously emit fluorescence. When a specific mutation is present, the mutation sequence hybridizes with the corresponding probe on the QD to form a double-stranded structure, leading to QD aggregation and subsequent fluorescence quenching. This method enables simultaneous detection of two mutations in clinical samples at 10 ng/μL (Figure 4B), with a detection limit as low as 0.01% (Kang et al., 2012).

CdTe QDs are known for their exceptional robustness (Farahmandzadeh et al., 2024; Yang et al., 2019). CdTe QDs also exhibit tunable fluorescence properties upon interaction with different nucleotides, including emission wavelength shifts (red- or blue-shift) and fluorescence quenching (Siegberg and Herten, 2011). These spectral changes allow for the differentiation of specific DNA sequences. Building on this principle, Moulick et al. developed an innovative approach for detecting DNA presence, damage, and mutations. They successfully identified the target DNA in extracts of human cancer cells (PC3) and normal cells (PNT1A) at concentrations as low as 500 pM (Moulick et al., 2017).

#### 3.2.2 UCNP-based fluorescence detection

Upconversion nanomaterials (UCNPs), a class of nanomaterials capable of converting low-energy light (infrared) into higher-energy light (visible or ultraviolet), have gained significant attention in bioimaging and spectral analysis (Mohan and Poddar, 2021). Kumar et al. designed an oligonucleotide sensor based on UCNPs (donor) and TAMRA fluorescent dye (acceptor) to detect the AT single-nucleotide mutation associated with sickle cell disease. This sensor captures the target DNA through a sandwich hybridization approach and triggers energy transfer based on the luminescence resonance energy transfer (LRET) mechanism. The method enables highly sensitive detection of the pathogenic AT mutation in sickle cell disease, achieving a detection limit as low as 120 fM (Kumar et al., 2009). Sensitive detection of DNA can also be achieved by tuning the excitation and emission wavelengths of UCNPs. In this approach, a near-infrared (NIR) fluorescent nanoprobe is constructed through complementary pairing of surface-modified DNA between UCNPs and silver nanoclusters (AgNCs). Experimental results demonstrated that the probe could detect *KRAS* gene mutations within a concentration range of 5 pM–1000 pM (Wang P. et al., 2014).

#### 3.2.3 Limitations and future optimization of fluorescence methods

Although fluorescence detection is more sensitive than colorimetric methods, background autofluorescence and nonspecific binding in complex samples still affect the signal-to-noise ratio (Izumi et al., 2011; Wang S. et al., 2023). In addition, quantitative results may be influenced by photobleaching, fluorescence quenching, and probe concentration, leading to insufficient reproducibility. By employing appropriate background suppression strategies, such as the use of MB or dual-probe recognition, nonspecific signals can be effectively reduced (Lee et al., 2024).

### 3.3 Electrochemical methods

Electrochemical methods usually affect the electrochemical signals through redox reactions, ion migration, or conductivity changes to achieve the detection of target molecules. Nanomaterials show great potential for use in electrochemical methods owing to their excellent electrical conductivity, large specific surface area, and good surface functionalization.

#### 3.3.1 Design and optimization of AuNP-based electrochemical methods

**FIGURE 5:**
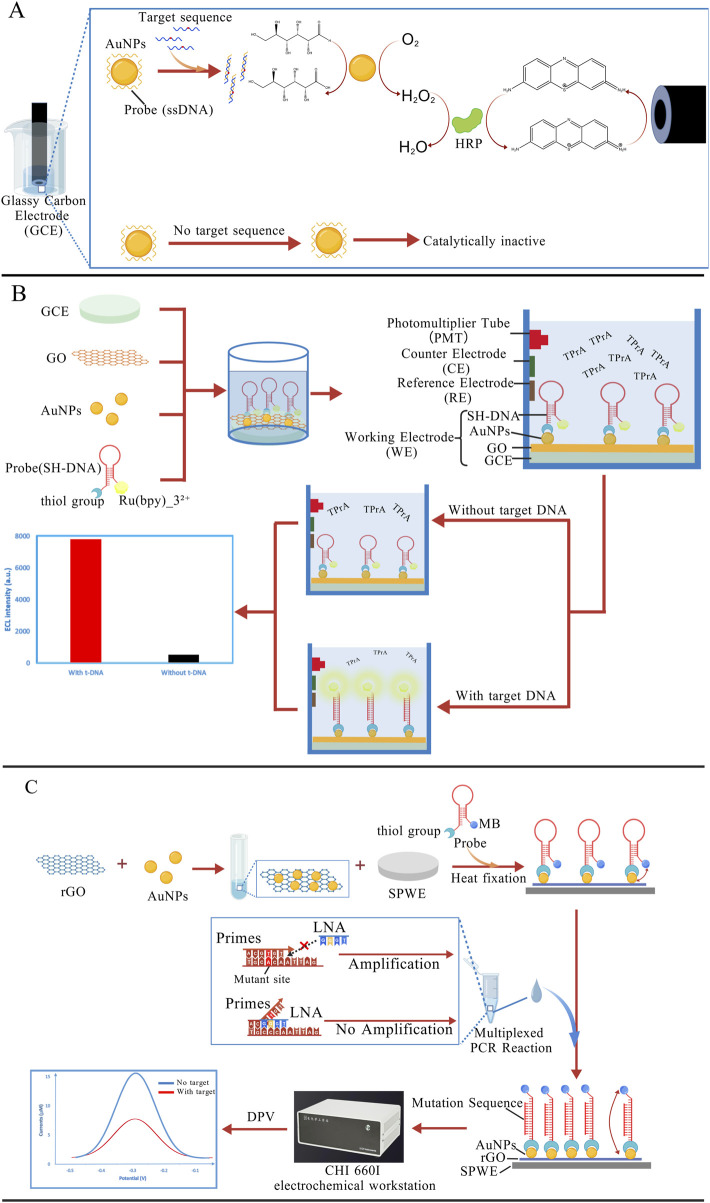
**(A)** AuNPs act as catalysts, and the catalytic activity is suppressed by the probes adsorbed on the surface. When the target DNA is present, the probes dissociate from the AuNP surface, thereby allowing the AuNPs to initiate a redox cascade reaction (Chen et al., 2014); **(B)** On the GCE, GO can quench the ECL of the Ru (bpy)_3_^2+^/tripropylamine (TPrA) system. In the presence of the target sequence, the hairpin probe structure unfolds, leading to the removal of GO from the electrode surface. As a result, the ECL signal is recovered (Huang et al., 2015); **(C)** Under the action of the LNA probe, the mutant sequence is selectively amplified. When the PCR products are introduced into the electrochemical sensor, the probe hairpin structure is opened, moving the MB molecule away from the electrode surface and resulting in a decrease in the electrochemical signal (Wang et al., 2022). The figures are created with BioGDP.com.

AuNPs have become a popular choice in building electrochemical biosensors. By coating electrode surfaces with AuNPs, these sensors can boost DNA hybridization signals, making them more effective at detecting base mismatches (Zhang and Zhang, 2010), especially AG mismatches. Compared to conventional sensors, AuNP-modified sensors exhibit significantly enhanced sensitivity, achieving detection limits in the nanomolar range (Wang Q. et al., 2011; Zhang R.-Y. et al., 2005). In AuNP-based mutation detection systems, the immobilization density of DNA probes critically governs both sensitivity and specificity. Conventional methodologies relying on thiol-Au covalent bonding are constrained by steric hindrance and inter-probe electrostatic repulsion, leading to suboptimal probe loading density and heterogeneous distribution. To enhance DNA probe attachment density, recent advancements incorporate vertically aligned multi-walled carbon nanotubes (MWCNTs) synergistically with AuNPs on modified electrodes. Experimental validation has demonstrated favorable detection sensitivity for sequence-specific hybridization events related to *TP53* gene detection, monitored via electrochemical impedance spectroscopy (EIS) (Fayazfar et al., 2014). In addition, current strategies predominantly immobilize AuNPs onto electrode surfaces via chemical bonding or physical adsorption. These strong interactions often result in permanent occupation of surface-active sites and irreversible alterations to the crystalline structure of AuNPs, severely compromising their reusability. To overcome this limitation, Chen’s team has proposed an electrode modification-free detection system in which AuNPs are directly used as the electrolyte. In this system, AuNPs act as catalysts whose activity is suppressed by the adsorption of ssDNA. The introduction of target DNA induces hybridization with the probe, resulting in desorption from the AuNP surface. The restored AuNPs catalyze the oxidation of glucose, initiating a cascade of redox reactions involving horseradish peroxidase (HRP) and the redox indicator thionine. The electrochemical signal is subsequently measured using a glassy carbon electrode (GCE) (Figure 5A). The system allows direct reuse by simply replacing the electrolyte solution for subsequent experiments (Chen et al., 2014).

#### 3.3.2 GO-based electrochemical methods

Other nanomaterials have also demonstrated comparable performance in electrochemical DNA sensing platforms (Hu et al., 2011; Mehrgardi and Ahangar, 2011; Muti et al., 2010; Skotadis et al., 2016). Graphene oxide (GO), one of the most versatile nanomaterials, has found widespread use in sensor development, electrochemical systems, and energy storage applications. Notably, GO efficiently and stably quenches the electrochemiluminescence (ECL) of the tris(2,2′-bipyridine)-ruthenium (II) (Ru (bpy)_3_^2+^)/tripropylamine (TPrA) system when deposited onto GCEs. This quenching behavior is primarily attributed to GO’s oxygen-rich surface functional groups, limited electrical conductivity, and the two-dimensional structure of the GO sheets (McCall et al., 1999). Subsequently, Huang et al. exploited this phenomenon to engineer a novel biosensing platform by incorporating GO onto AuNP-functionalized GCEs via DNA probe self-assembly. The GO-mediated ECL quenching served as the core detection mechanism. The DNA probes maintained intimate contact with GO surfaces without target DNA, resulting in substantial ECL suppression. Hybridization with complementary targets induced probe detachment from GO, thereby restoring ECL signals (Figure 5B). Systematic characterization revealed a linear response to target DNA concentrations spanning six orders of magnitude (100 a.m.-10 pM) (Huang et al., 2015).

#### 3.3.3 QD-based electrochemical methods

QDs exhibit not only excellent optical properties but also favorable electron transport and storage capabilities, and even the fundamental property of improving electrode interfaces. Therefore, some research groups have combined QD with electrochemical methods for detection. In the electrochemical sensor designed by Mazloum-Ardakani et al., CdS QDs were utilized as the primary functional material to enhance the electroactivity of the electrode interface. This sensor demonstrated the successful application of high-sensitivity detection for the *ApoB100* R3500Q mutation (Mazloum-Ardakani et al., 2015). Yang et al. developed a highly sensitive ECL biosensor by employing CdS QDs labeled with thioglycolic acid (TGA) and AuNP-labeled hairpin DNA probes. In the absence of target DNA, FRET occurs when AuNPs are in close proximity to CdS QDs, quenching the ECL signal of the CdS QDs. In the presence of target DNA, the hairpin structure is opened, increasing the distance between AuNPs and CdS QDs, thereby reducing energy transfer and enhancing the ECL signal. This biosensor ultimately achieved precise detection of the *EGFR* T790M mutation, with a detection limit as low as 3.4 a.m (Yang et al., 2023). This approach presents a novel strategy for electrochemical processes incorporating QDs, which could similarly be extended to other QDs (Yang et al., 2021).

#### 3.3.4 Combining nanoparticles with amplification techniques

As electrochemical detection techniques improve, researchers are exploring novel strategies that integrate selective nucleic acid amplification techniques with nanoparticles (Lu et al., 2017; Yang et al., 2016). As an example, Wang et al. developed a detection platform that combines electrochemical sensing with LNA-mediated multiplex PCR. The system enables selective amplification of mutant *EGFR* genes (L858R, T790M), followed by detection of the amplified products using a screen-printed working electrode (SPWE) modified with reduced graphene oxide-gold nanoparticles (rGO-AuNPs). In the absence of target DNA, the probe folds into a hairpin structure that brings the methylene blue (MB) molecule close to the electrode surface. As an electroactive molecule, MB enables efficient electron transfer between the electrode and itself, generating a strong current signal. In the presence of the mutant sequence, the hairpin structure of the probe unfolds, causing the MB label at its end to move away from the electrode surface. This structural change leads to a decrease in the electrochemical signal (Figure 5C). Experimental results showed that this method could accurately detect rare mutations at just 0.05%, offering a sensitivity improvement of two orders of magnitude compared to traditional electrochemical methods (Wang et al., 2022). Owing to the signal amplification ability of nanoparticles and their applicability under different experimental conditions, nanoparticles are promising for improved diagnostics of several diseases.

#### 3.3.5 Limitations and future optimization of electrochemical methods

Electrochemical gene detection usually requires electrode modification with nanomaterials or functionalization (such as AuNPs or carbon nanotubes), which involves complex processes and high equipment demands. In addition, conformational changes of immobilized probes on the electrode surface and nonspecific adsorption can interfere with signal interpretation. By introducing novel electrocatalytic materials such as nanozymes or MOFs, the signal intensity and stability can be improved, thereby partially addressing these issues (Liu et al., 2024; Wang Y. et al., 2023).

### 3.4 Surface-enhanced Raman scattering (SERS)

Raman scattering signals arise from the inelastic scattering of light with matter. Although Raman signal intensity constitutes only about one-millionth of the incident light, when molecules are adsorbed onto nanoscale rough surfaces (such as AuNP, CuNP, or nanostructures), the Raman scattering signal is greatly enhanced, and even single-molecule detection can be achieved. The enhancement effect mainly originates from Localized Surface Plasmon Resonance (LSPR) and the chemical enhancement mechanism between the molecule and the metal surface. Owing to the unique molecular fingerprint characteristics of SERS, this technique enables highly sensitive detection of specific molecules at extremely low concentrations (Lyu et al., 2024).

**FIGURE 6:**
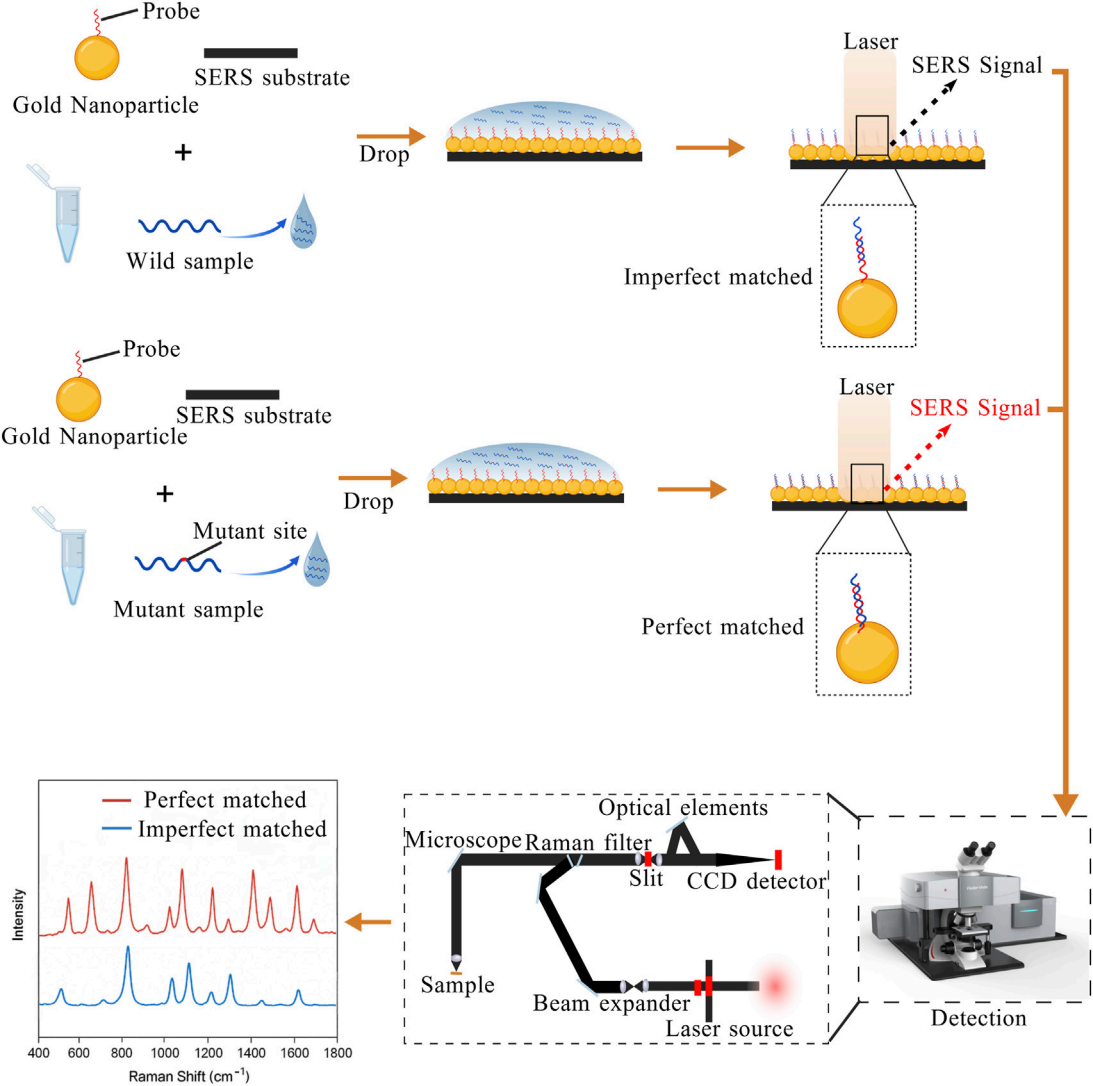
The probes on gold nanoparticles are partially complementary to the wild-type sequence but perfectly complementary to the mutant sequence, generating distinguishable SERS signals. The figure is created with BioGDP.com.

Detection of target molecules with SERS can be accomplished by using two main approaches. SERS can directly obtain structural information of molecules through the enhanced Raman signal of molecules that come close to the metal surface (Xie and Schlücker, 2013). Alternatively, SERS labels or nanolabels conjugated to recognition elements (e.g., antibodies) produce strong Raman signals in proximity to the target, enabling both qualitative and quantitative analysis in complex biological environments (De Silva Indrasekara and Fabris, 2015). Accordingly, the design and selection of nanomaterials are crucial in SERS experiments. In nucleic acid detection, SERS mainly utilizes the design of specific nucleic acid probes, which hybridize with target DNA or RNA. Probes designed on the metal surface form perfect matches with mutant sequences and imperfect matches with wild-type sequences, generating different SERS signals. This difference in signal constitutes the key principle of using SERS to detect point mutations, as shown in Figure 6. The proper design of nanostructured surfaces critically affects both the detection rate and the detection limit of mutation sequences.

#### 3.4.1 Combining SERS with PCR

Li et al. employed a modified sodium citrate reduction method to synthesize AgNP colloids with irregular surface roughness, which facilitated the formation of “hot spots”. Subsequently, the addition of spermine acted as both an aggregating agent and a neutralizer of the DNA backbone, leading to further aggregation of AgNP and the formation of additional hot spots, thereby significantly enhancing the SERS signal. In addition, they combined SERS with multiplex PCR to simultaneously amplify multiple mutation types in a single reaction. The specifically designed nucleic acid probes produced distinct SERS signals when hybridized with different sequences, and the relative peak intensities in the SERS spectra enabled discrimination among various nucleic acid sequences. Using this approach, they successfully achieved simultaneous detection of multiple hotspot point mutations in genes such as *KRAS* and *PIK3CA* (Li et al., 2017; Li et al., 2018). In contrast to conventional PCR, asymmetric PCR (Asy-PCR) adjusts the ratio of forward and reverse primers to preferentially generate ssDNA. A research team developed a highly specific Asy-PCR/SERS method to detect *KRAS* G12 V mutation. The researchers chose to functionalize AuNPs and then conjugated them with the Raman reporter molecule DTNB and mutation-specific DNA probes to form SERS nanotags. Subsequently, PCR products were hybridized with the tags via heating and annealing, the target complexes were enriched using streptavidin-coated magnetic beads, and characteristic peaks were detected with a Raman spectrometer. This method can be used for the detection of *KRAS* G12V mutations at just 0.1% in the presence of non-target sequences (Lyu et al., 2021). However, a limitation of this approach lies in the potential self-folding of ssDNA products generated by Asy-PCR, which may form unintended secondary structures (e.g., hairpins), thereby reducing hybridization efficiency with specific probes. Although optimized PCR designs allow for the simultaneous amplification of multiple DNA targets and improve detection throughput, they remain limited by the intrinsic complexity of the amplification process. To address this, Wu et al. proposed an amplification-free SERS-based strategy. They used Au@Ag core-shell nanorods (Au@Ag NRs) to prepare a highly sensitive and uniform SERS substrate, yielding an enhancement factor of 1.85 × 10^5^. By this approach, they successfully distinguished mutant from wild-type *KRAS* genes with a detection limit of 50 fM (Wu et al., 2019).

#### 3.4.2 Limitations and optimization of SERS-based point mutation detection

Although SERS has shown great promise in boosting sensitivity, especially combined with other techniques, traditional approaches still face a few notable hurdles in DNA analysis. For instance, signal instability frequently results from interference caused by impurities and nanomaterials with low surface enhancement factors, with thymine (T)-derived signals being particularly susceptible (Wang H. et al., 2014). To address these issues, researchers have introduced thiosulfate-modified AgNPs, which not only eliminate the unwanted effects of impurities but also help maintain the integrity of DNA signals (Zeng et al., 2021; Zhang et al., 2022). Another limitation arises from the intrinsically small scattering cross-sections of conventional Raman markers. Li et al. engineered a hybrid sensing platform which integrates silver nanorice antennae with gold triangular nanoprism arrays (SNA-GTNA) for the detection of HBV DNA. This nanoarray-coupled architecture demonstrated a remarkable SERS signal amplification exceeding four orders of magnitude compared to planar gold film substrates, accompanied by an extended electromagnetic field enhancement spanning hundreds of nanometers (Li et al., 2013). Notably, while classical theory posits that Raman signal intensity diminishes with increasing distance between reporter molecules and metallic substrates, emerging evidence reveals counterintuitive enhancement phenomena in SERS-based sandwich-structured DNA sensors. Specifically, certain configurations exhibit stronger signals when reporters are positioned farther from the metallic surface. This spatial dependency introduces new design considerations for optimizing SERS-based nucleic acid detection architectures (Pyrak et al., 2023).

SERS technology demonstrates exceptional sensitivity and resolution not only in genetic-level analyses but also in disease subtyping, pathogen identification, and viral infection detection (AlSafadi et al., 2024; Chen et al., 2025; He et al., 2024; Ye et al., 2022). Although nanoparticle-based detection technologies possess great potential for sensitivity and specificity, the toxicity of such particles is still nonnegligible. The exploration of nanoparticle biocompatibility and biodegradability is of priority to future research. Thus, analytical performance must be balanced against potential biological hazards to ensure safe diagnostic applications (Brunelli et al., 2024; Ruijter et al., 2023).

## 4 Nucleic acid analogs-based mutation detection

### 4.1 Peptide nucleic acid (PNA)

PNA is a synthetically engineered molecule. Unlike DNA, which features a negatively charged phosphodiester backbone, PNA possesses a neutral pseudo-peptide backbone. This structural distinction endows PNA with several unique properties. On one hand, the peptide backbone renders PNA electrically neutral. The lack of electrostatic repulsion enables PNA to exhibit significantly higher binding affinity to complementary DNA than conventional DNA/DNA duplexes (Almarsson and Bruice, 1993; Nielsen, 1997). On the other hand, PNA demonstrates high resistance to common nucleases and proteases (Yagita et al., 2020), maintaining excellent stability both *in vitro* and *in vivo* (Rose et al., 2019). Owing to its high binding specificity and affinity for target DNA or RNA, PNA has been widely applied in nucleic acid detection platforms.

#### 4.1.1 Combining PNA with PCR

**FIGURE 7:**
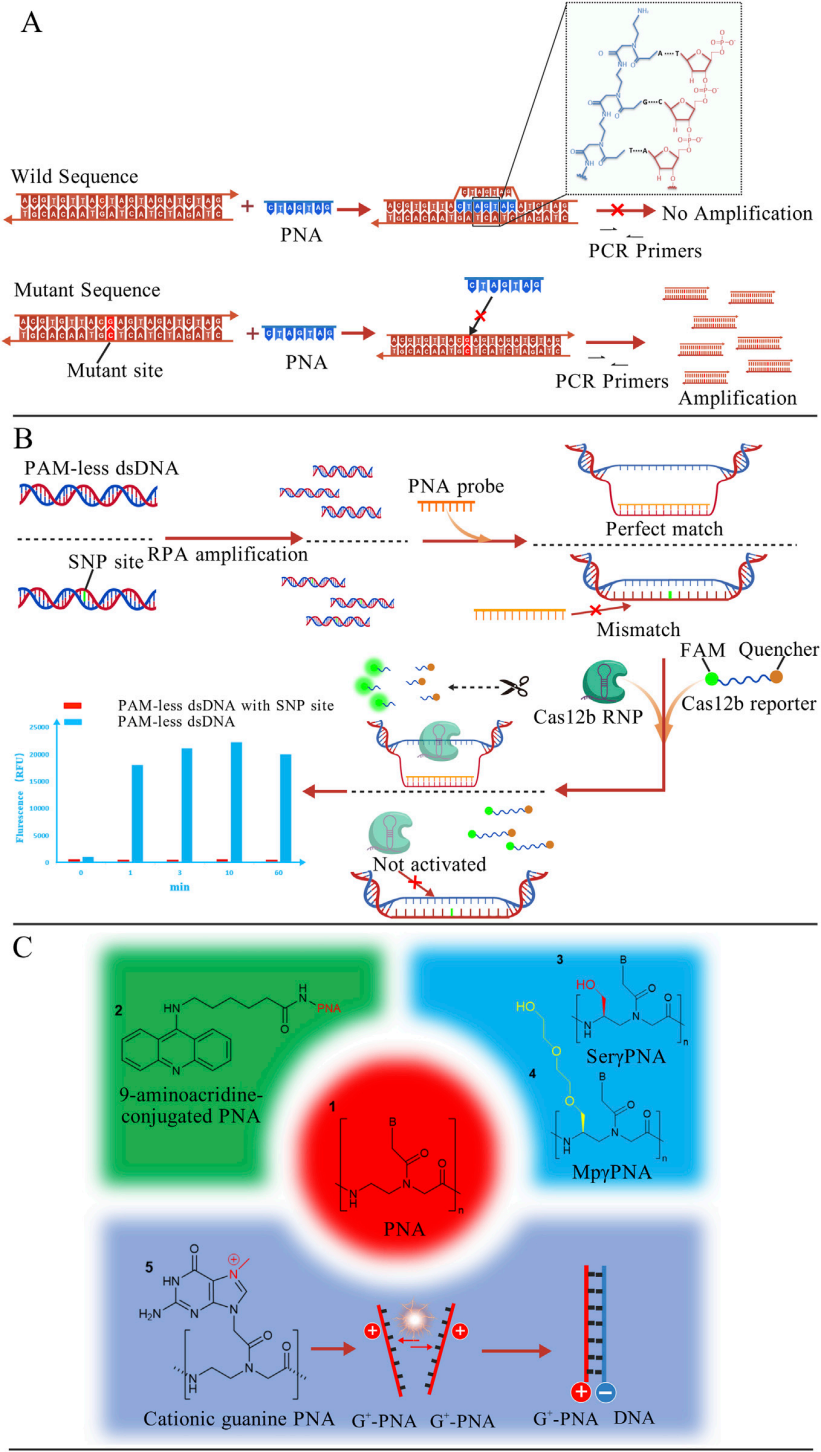
**(A)** PNA probes invade wild-type dsDNA, preventing its subsequent amplification. Mutant DNA, which cannot be invaded, can thus be amplified and detected. The binding mode of PNA with DNA is shown in the figure (Fouz and Appella, 2020); **(B)** PNA probes invade PAM-less dsDNA and generate a ssDNA window. Subsequently, Cas12b RNP and reporter molecules are introduced. The exposed ssDNA activates the trans-cleavage activity of Cas12b, which cleaves the reporter molecule and generates a strong fluorescence signal. PAM-less dsDNA with SNP site cannot be invaded and fails to activate Cas12b cleavage activity (Jiang et al., 2023); **(C)** Structural formula of PNA with specific molecular modifications (Bentin and Nielsen, 2003; Hibino et al., 2020; Oyaghire et al., 2023). The figures are created with BioGDP.com.

Due to its resistance to DNA polymerase, PNA has been combined with PCR to develop PNA-mediated PCR clamping technology (Fouz and Appella, 2020). Its principle is shown in Figure 7A, PNA probes can invade wild-type dsDNA and bind tightly to it, thereby preventing subsequent PCR amplification. In contrast, due to the presence of the mutation site, the probe cannot invade mutant dsDNA, allowing the mutant sequence to be normally amplified. A research team employed this method to evaluate the feasibility of plasma cell-free DNA (cfDNA) as an alternative specimen for detecting *EGFR* mutations in NSCLC patients. Among 40 patients with confirmed tumor tissue mutation status, 35 were identified as carrying *EGFR*-activating mutations (Kim et al., 2013). Although this approach successfully achieved partial non-invasive detection, its sensitivity remains suboptimal and requires further optimization. Rosso and co-workers integrated fluorescence *in situ* hybridization (FISH) with PNA-PCR by employing fluorescence-labeled PNA probes to detect *BCR-ABL* T315I mutations in two subtypes of chronic myeloid leukemia (CML). This approach achieved a detection sensitivity of 0.5% mutant alleles in mixed samples (Rosso et al., 2015).

#### 4.1.2 Combining PNA with protease

Compared to DNA, PNA exhibits greater structural flexibility (Chhetri et al., 2022), allowing it to adopt multiple conformations. This property enables PNA to interact with a broad range of biomolecules, including enzymes (Saikia et al., 2021). Jiang et al. developed a PNA-based universal detection toolkit (PNA-Pdx) that integrates the high specificity of PNA probes with CRISPR-Cas12b systems, as shown in Figure 7B. Relying on its high affinity, PNA invades and locally unwinds wild-type dsDNA, forming a ssDNA window. Subsequently, the Cas12b-sgRNA complex and fluorescent reporter molecules labeled with a fluorophore and quencher are introduced. If the exposed single-stranded sequence is fully complementary to the sgRNA, the trans-cleavage activity of Cas12b is activated, cleaving the reporter and generating a strong fluorescence signal. In the presence of an SNP mismatch, however, the probe cannot effectively invade, resulting in markedly reduced Cas12b activity and quenched fluorescence, thereby enabling precise SNP discrimination. This platform successfully detected diverse mutation types including AG, GC, and TA substitutions with a sensitivity of 300 pM. The methodology not only enhanced detection sensitivity but also overcame the PAM dependency constraints inherent to conventional CRISPR technologies (Jiang et al., 2023).

#### 4.1.3 Limitations and optimization of PNA probe

Although conventional PNA is theoretically capable of sequence-specific binding to DNA or RNA, its practical binding efficiency is often suboptimal. Researchers typically address this by modifying PNA with auxiliary molecules at specific sites to enhance binding affinity (Figure 7C). For instance, acridine-conjugated PNAs exhibit binding affinities 20–150-fold higher than those of unmodified PNAs (Bentin and Nielsen, 2003). Further structural optimizations, such as γ-position modifications with N7-methylguanine (G+), hydroxymethyl and diethylene glycol, have been demonstrated to enhance PNA binding capacity to varying degrees (Hibino et al., 2020; Oyaghire et al., 2023). Additionally, conventional PNAs exhibit inherent challenges including poor water solubility and inefficient cellular internalization (Gasparello et al., 2019). To overcome these barriers, Aiba et al. conjugated nuclear localization signal (NLS) peptides to pseudo-complementary PNA (pcPNA) (Aiba et al., 2015). Experimental validation revealed that NLS-pcPNA rapidly formed invasion complexes with dsDNA even at low concentrations and under high-salt conditions (100 mM NaCl). Moreover, the predominant synthesis of PNA via solid-phase peptide synthesis (SPPS) incurs high production costs, restricting its widespread adoption. Researchers have reduced the cost of PNA synthesis by employing physicochemical strategies such as utilizing low-cost commercial fluid-handling components (Kallmyer et al., 2020), incorporating safety-catch protecting groups (Nandhini et al., 2023), and implementing ultrasound-assisted synthesis (Del Bene et al., 2023). With continued advancements in synthetic technologies, it is anticipated that more cost-effective synthesis approaches will emerge in the future.

### 4.2 Locked nucleic acids (LNA)

LNA is a chemically modified nucleic acid analog characterized by a unique structure. This structure is formed by a methylene bridge linking the 2′-oxygen and 4′-carbon atoms of the ribose ring. This modification locks the ribose in a C3′-endo conformation, significantly enhancing its binding affinity for complementary DNA or RNA strands (Egli et al., 2001; Petersen et al., 2002). Compared to regular DNA, LNA provides a 2 °C–8 °C increase in thermal stability (ΔT_m_) for each incorporated monomer (Owczarzy et al., 2011), along with better discrimination against base mismatches (Mishra et al., 2013). Owing to these properties, LNA has emerged as an optimal candidate for the design of high-sensitivity nucleic acid probes.

#### 4.2.1 Design and optimization of LNA probes

**FIGURE 8:**
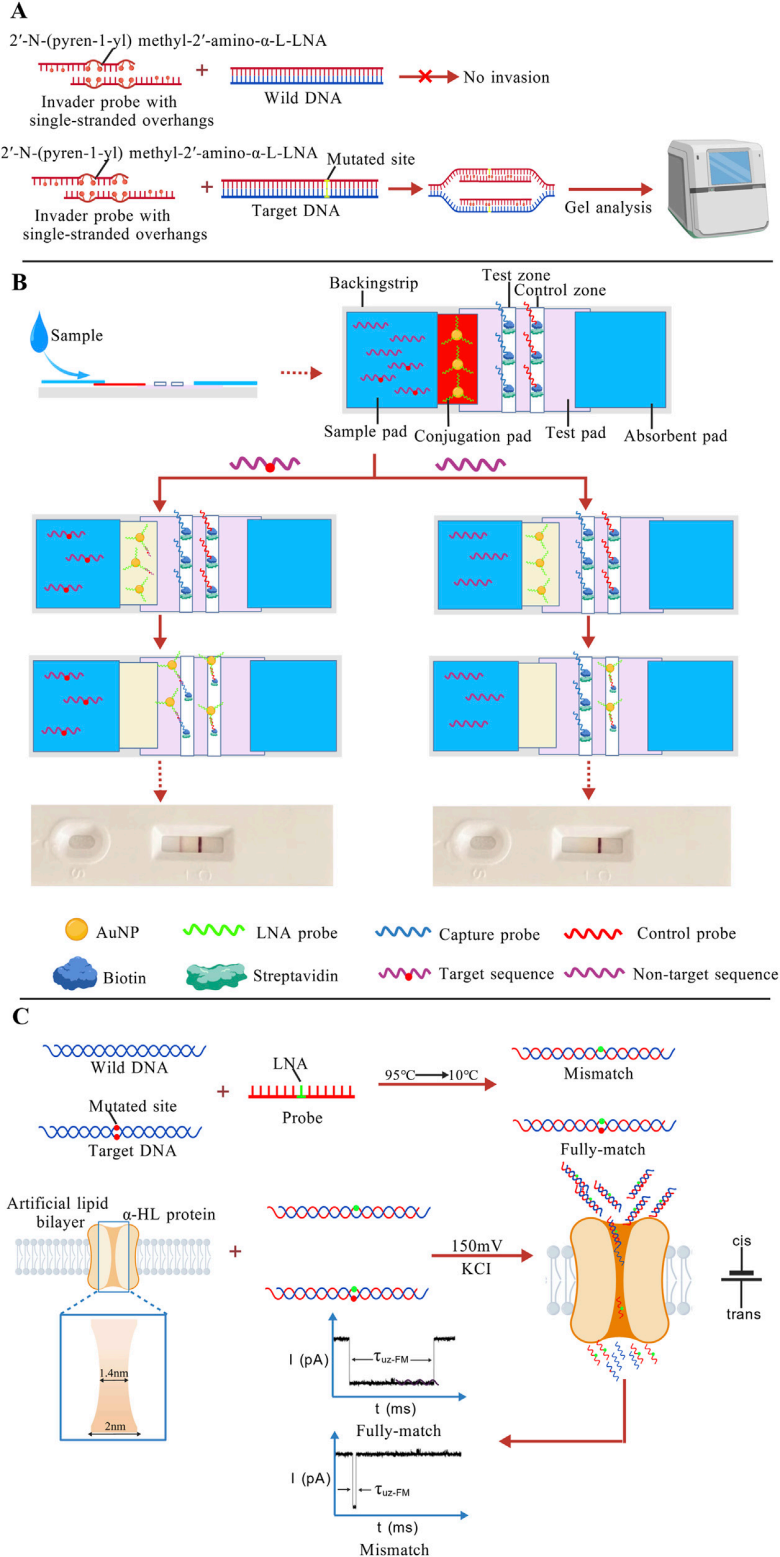
**(A)** Probes with single-stranded overhangs can exclusively invade mutant DNA without affecting wild-type DNA, ultimately allowing differentiation through gel electrophoresis results (Adhikari et al., 2022); **(B)** The target sequence migrates from the sample pad to the conjugation pad via capillary action, where it binds to LNA-modified AuNP probes to form a complex. A part of the complex is captured by the capture probe. Excess gold nanoparticle probes are captured by complementary probes at the C line, resulting in color development at the T line and C line. The appearance of two lines indicates a positive detection result. When the target sequence is absent in the sample, no complex is formed. Only the LNA probes are captured and visualized at the C line, producing a single pink band, which indicates a negative detection result (Rastogi et al., 2012); **(C)** The LNA probe is fully complementary to the mutant DNA but mismatched with the wild-type DNA. Under an applied voltage, mismatched duplexes dissociate, and translocate rapidly through the nanopore, whereas the perfectly matched duplex passes through more slowly (Tian et al., 2018). The figures are created with BioGDP.com.

Traditional invader probes are short, blunt-ended dsDNA. By incorporating intercalating agents such as 2′-O-(pyren-1-yl)methyl-RNA monomers arranged in a specific zipper configuration, these probes generate “energetic hotspots”. These hotspots weaken the stability of the DNA duplex by disrupting base-pair stacking. However, conventional Invader probes exhibit poor efficiency for long DNA fragments and GC-rich sequences, and show dependency on specific ionic strength and pH conditions. Building upon this framework, researchers developed a novel dsDNA-targeting probe, termed Invader LNA, by incorporating 2′-N-(pyren-1-yl)methyl-2′-amino-α-L-LNA monomers. This probe maintains robust mismatch discrimination capability even under high-salt (710 mM Na^+^) and physiologically relevant ionic strength conditions (Sau et al., 2010). Moreover, incorporating a 3-6 nucleotide single-stranded overhang at the 5′ end of the probe shortens the duplex region, further promoting strand separation. The new designed invasive LNA probes can invade mutant sequences, and be detected by gel analysis (Figure 8A). Experimental results demonstrated that this design further enhances both the sensitivity and specificity of the Invader LNA probes (Adhikari et al., 2021; Adhikari et al., 2022).

#### 4.2.2 Combining LNA with PCR

Similar to PNA, the high binding affinity and specificity of LNA probes enable the selective suppression of wild-type DNA amplification during PCR, allowing for the detection of mutations at frequencies as low as 0.1% (Ishige et al., 2016; Warshawsky and Mularo, 2011). Furthermore, a synergistic approach combining both PNA and LNA has been developed. In this strategy, fully complementary PNA probes stably bind to and block the amplification of wild-type DNA. Meanwhile, LNA-modified primers complementary to mutant sequences facilitate selective amplification of mutant DNA. This method achieved rapid *KRAS* G12D and G12V mutation detection by loop-mediated isothermal amplification (LAMP) at 65 °C. The assay demonstrated a detection sensitivity exceeding 0.1%, surpassing that of conventional sequencing and traditional PNA-clamping PCR (Itonaga et al., 2016). The biocomplex formed by the joint application of PNA and LNA interacting with target DNA exhibits enhanced stability and specificity (Lundin et al., 2005).

#### 4.2.3 Combining LNA with nanotechnology

Among various nanomaterials, AuNPs are the most commonly used in combination with LNA (Lin et al., 2013a; 2013b). The excellent mismatch discrimination capability of LNA, together with the stability and signal amplification properties of AuNPs, often enables lower detection limits. For instance, Shiva et al. developed a lateral flow test strip that integrates LNA-modified probes with gold nanoparticles for the detection of DNA specific to the foodborne pathogen *E. coli* O157:H7. After the target DNA sample binds with the LNA-modified AuNP probes on the conjugation pad to form a complex. A part of the target DNA is specifically captured by the immobilized capture probe in this zone, thereby aggregating the AuNPs on the test line (T line) to form a visible pink band. Excess AuNP probes continue to flow further to the control line (C line), where they are captured by another complementary probe, forming a second pink band-resulting in the appearance of two lines. If the target DNA is absent, the AuNP probes cannot form the complex. However, these free probes continue to flow to the C line, where they are immobilized, forming a single pink band (Figure 8B). This method achieved a detection limit as low as 0.4 nM (Rastogi et al., 2012). LNA can not only work synergistically with traditional inorganic nanomaterials such as AuNPs and MNPs (Wang K.-H. et al., 2024), but also be involved in the applications of biological nanosensors. In one study, researchers embedded α-hemolysin (α-HL) protein nanopores into an artificial phospholipid membrane to construct a nanopore-based biosensor. The narrowest region of the nanopore channel measures approximately 1.4 nm in diameter, allowing only ssDNA to pass through. The designed LNA probes partially hybridize with the wild-type sequence and fully complementary with the sequence containing a mutation site. When a specific voltage is applied to the sensor, the partially complementary DNA sequences rapidly dissociate due to reduced double-strand stability, and the dissociated ssDNA can pass through the nanopore within a short time. In contrast, the fully matched sequences require more time to pass (Figure 8C). By analyzing the current signals and translocation duration, the system can distinguish between wild-type and mutant sequences (Tian et al., 2018).

#### 4.2.4 Limitations and future optimization of LNA probes

However, LNA remains more costly than conventional probes (Hansen et al., 2003; Pfundheller et al., 2005). The incorporation positions of LNA also require precise optimization (Laxton et al., 2011), and extensive LNA modifications may induce structural rigidity in the probes (Crinelli, 2002; Ferreira et al., 2021). Additionally, the potential toxicity risks associated with LNA probes warrant further investigation, as high concentrations of LNA-modified oligonucleotides might trigger immune responses and hepatotoxic effects (Romero-Palomo et al., 2021; Swayze et al., 2007). Researchers have developed LNA structure-toxicity prediction tools to assist in the optimization of LNA design and reduce the risk of toxicity (Burdick et al., 2014).

## 5 Pure nucleic acid probe-based detection

In recent years, an increasing number of studies have focused on point mutation detection by exploiting the intrinsic higher-order structures and dynamic intermolecular reactions of nucleic acids themselves (Lahiri et al., 2019; Zhang et al., 2023). This approach does not rely on exogenous components such as enzymes or nanomaterials, but rather achieves highly sensitive discrimination of single-base differences through conformational transitions of nucleic acids at the secondary or higher structural level.

**FIGURE 9:**
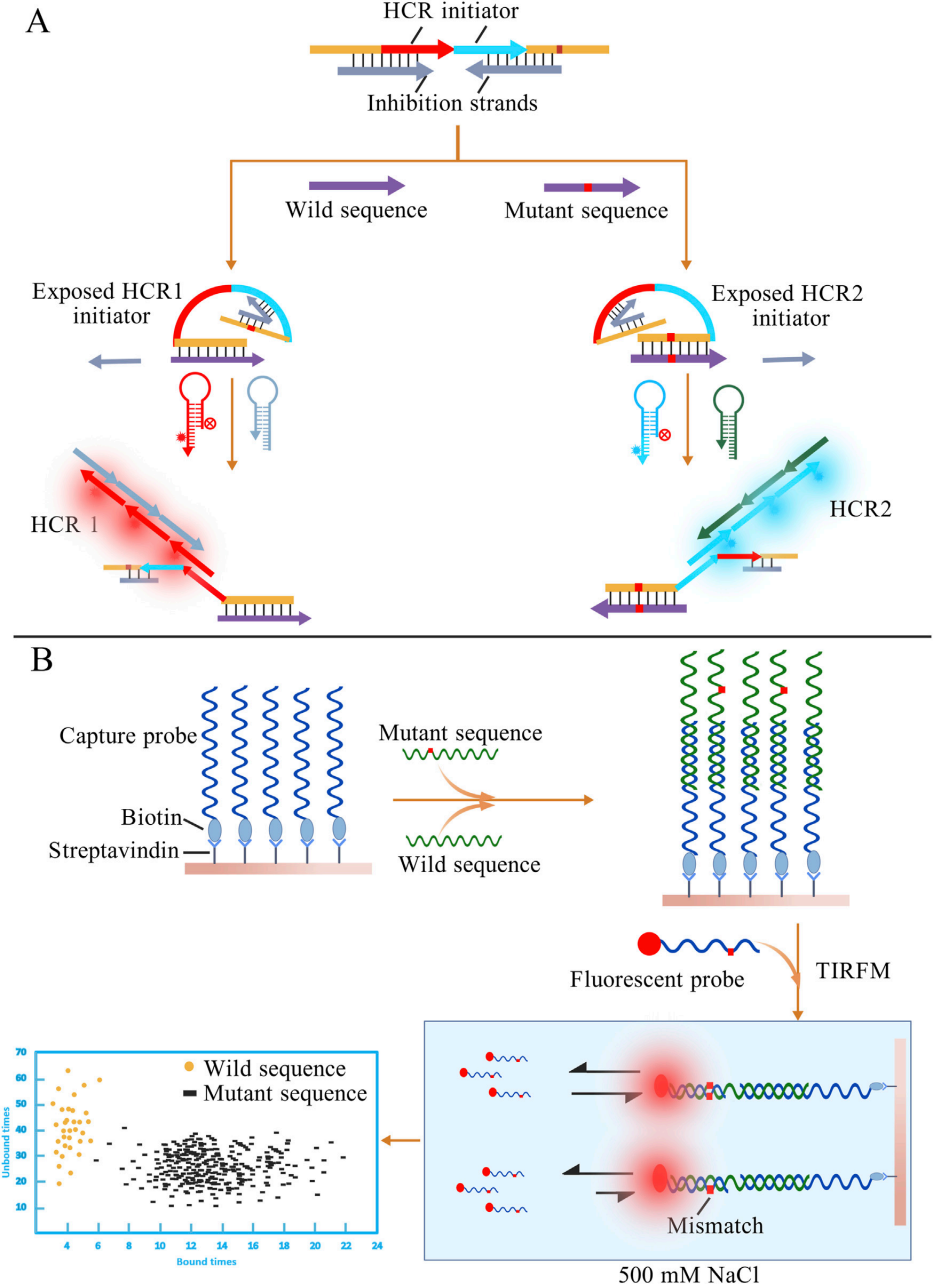
**(A)** Different target sequences displace the corresponding inhibitory strands, thereby exposing distinct HCR initiators. The presence of wild-type or mutant sequences can then be determined based on the resulting HCR fluorescence signals. **(B)** Excess capture probes first hybridize with the nucleic acid sequences in the sample. After adding fluorescent probes, fully matched sequences exhibit longer binding durations with the probes. The presence of mutant sequences is determined by observing the duration of fluorescent signals under TIRFM. The figures are created with BioGDP.com.

Based on nucleic acid kinetics, Wu et al. proposed and validated a novel terminal self-competitive nucleic acid probe (TSCP). The entire probe consists of one long strand and two short inhibitory strands. The long strand contains complementary sequences to both the wild-type and mutant genes, as well as two distinct HCR initiators. In the absence of the target, the probe tightly binds to the inhibitory strands, thereby “locking” the HCR initiators. When the sample is introduced, the wild-type and mutant sequences replace their respective inhibitory strands and hybridize with the probe, simultaneously exposing the HCR initiators (Figure 9A). Subsequent fluorescence signals indicate the presence of the corresponding sequences, enabling accurate detection at concentrations as low as the nM level (Wu et al., 2025). However, in traditional competitive probe systems, it is often difficult to simultaneously achieve high sensitivity and specificity. The length and concentration of the blocking strand usually exhibit a negative correlation, making the optimization process cumbersome. Chen et al. proposed the 4-way Strand Exchange LEd Competitive DNA Testing (SELECT) system, which effectively addresses this issue by allowing the probe to bind to the target sequence and generate a signal without requiring complete dissociation from the inhibitory strand. This system successfully detected *KRAS* G12D and G13D mutations in ovarian cancer patient samples, with a detection limit of 0.1% (Chen et al., 2019). Conventional nucleic acid probes are generally designed to be long, since shorter probes tend to destabilize probe-target duplexes and cause transient dissociation. Interestingly, Su et al. turned this inherent limitation into an advantage by establishing a detection method that leverages the transient binding and dissociation of short DNA probes (Su et al., 2017). In high-salt conditions, short fluorescent probes are driven to associate with the target sequence, producing detectable fluorescence upon hybridization. However, even a single-base mismatch is strongly penalized under such high-salt environments. By monitoring the fluorescence duration with total internal reflection fluorescence microscopy (TIRFM), mutant and wild-type sequences can be effectively distinguished. A schematic diagram of this method is shown in Figure 9B. Such methods that differentiate molecules based on kinetic differences are sometimes referred to as “Kinetic Fingerprinting”.

Beyond reliance on intrinsic nucleic acid kinetics, certain research groups have introduced chemical modifications (such as pyrene and 2′-O-methyl RNA) into probe design to enhance structural stability and detection sensitivity (Kholodar et al., 2013; Zhou et al., 2018). Meanwhile, other groups have sought to integrate nucleic acid probes with complementary analytical technologies. Zhang et al. combined catalytic hairpin assembly (CHA), FRET, and toehold-mediated strand displacement (TMSD) to develop a dual-base mismatch strategy. By introducing an extra mismatch, the system could distinguish WT from MT with high sensitivity, reaching a detection limit of 4.3 fM (Zhang et al., 2024).

In addition to the limitations mentioned above, pure nucleic acid probe-based detection also suffers from limited anti-interference capability and insufficient dynamic regulation, which lead to reduced detection efficiency in complex biological samples. However, with a deeper understanding of nucleic acid kinetics, energy differences, and free energy landscapes, it is believed that highly specific and selective enzyme-free nucleic acid probes will be designed in the future for the direct detection of point mutations.

## 6 From laboratory to clinic

Validation using clinical samples represents the next critical step for most nucleic acid probe-based detection methods, which will establish a solid foundation for their comprehensive advancement into clinical testing. However, the vast majority of the experimental strategies mentioned above remain confined to the laboratory stage, utilizing either genetically extracted materials from self-cultured cells or artificially synthesized nucleotide fragments. Substantial differences exist between real clinical samples and these idealized specimens, as complex background factors may significantly compromise detection efficiency. Thus, transitioning success in experimental models to success in clinical samples constitutes a major challenge for current nucleic acid probe-based detection technologies.

However, several studies have successfully achieved validation using real clinical samples. Examples include the NAVIGATER system proposed by Song et al. (2020), the AuNP-based colorimetric method employed by Bai et al. (2020), and so on (Table 2). Although the detection sensitivity was slightly lower than that observed with synthetic ssDNA, these approaches still achieved considerable detection limits. Nevertheless, critical issues must be addressed before large-scale clinical application and integration into clinical decision-making can be realized. These include the inherent stability of enzymes, the biotoxicity of nanomaterials, the water solubility of PNA probes, and challenges related to their delivery.

**TABLE 2:** A summary of technologies applicable to clinical sample detection.

Method	Target	Sample	References
DASH	*KRAS* G12D, rRNA	CSF	Gu et al. (2016)
Cas9-AFLPA	*EGFR*	Tissue	Jia et al. (2018)
PAND	*BRCA1*	Serum	He et al. (2019)
Ago-GFET	CfDNA	Plasma	Kong et al. (2024)
NAVIGATER	*KRAS*	Blood	Song et al. (2020)
PCR-LDR-qPCR	*TP53*	Plasma and tissue	Ruiz et al. (2020)
Cas14a-LCR-RCA	*BRAF* V600E	FNA	Shi et al. (2022)
DNAzymes-LCR	β-globin gene	Blood	Zhou et al. (2013)
DNAzymes-FEN1-CHA	*KRAS*	Serum	Li et al. (2022)
DNAzymes-CHA-CL	*MAPT*	Saliva	Xu et al. (2017)
AuNPs-assisted colorimetry	*PIK3CA*	Blood	Bai et al. (2020)
QD-FRET DNA nanosensor	*KRAS*	Tissue	Zhang et al. (2005a)
QD-assisted dual-color fluorescence assay	*KRAS*	Tissue	Yeh (2006)
GO-GCE electrochemical sensor	DNA	Serum	Huang et al. (2015)
QD electrochemical sensor	*ApoB100* R3500Q	Blood	Mazloum-Ardakani et al. (2015)
CdS QD-ECL sensor	*EGFR* T790M	Plasma	Yang et al. (2023)
rGO-AuNP-ePCR	*EGFR*	Tissue	Wang et al. (2022)
PCR-SERS	*PIK3CA* E542K	Plasma	Li et al. (2018)
Asy-PCR-SERS	*KRAS* G12V	Tissue	Lyu et al. (2021)
PNA-PCR-FISH	*BCR-ABL* T315I	Bone marrow	Rosso et al. (2015)
LNA-MNPs-SERS	hsa-miR-200a-3p	Plasma	Wang et al. (2024b)
PNA-Pdx	SARS-CoV-2 genome	Nasopharyngeal swabs	Jiang et al. (2023)
SELECT	*KRAS* G12D, G13D	Blood	Chen et al. (2019)
AI-RCA-RPA	*KRAS* G12V	Tissue	Sebuyoya et al. (2025)
AI-ddPCR	*BRAF* V600E	Tissue	Colozza-Gama et al. (2021)

Currently, nucleic acid probe-based methods that have been truly successfully applied to clinical testing are still predominantly PCR-based detection systems, such as fluorescent PCR and digital PCR (Ryncarz et al., 1999; Stamey et al., 2001). Commercial PCR kits targeting common mutation sites are readily available on the market and have achieved successful commercialization. For patients, this eliminates the need to endure lengthy and complex processes like Sanger sequencing or NGS. Moreover, compared to sequencing, precise PCR detection offers significantly reduced costs. The commercial success of PCR-based assays is also helping drive the development of more nucleic acid probe-based detection technologies, though several limitations remain. PCR-based methods require complicated thermal cycling procedures, during which enzymes are still indispensable. The development of simpler and faster approaches, ideally operable at room temperature, remains a key direction for future research. Nucleic acid probe-based detection also has a major advantage: once a simple and efficient detection strategy is established, it can be readily adapted to other rare sites simply by altering the probe sequence. Nevertheless, specific kinetics and GC content still need to be taken into consideration.

While nucleic acid probe-based approaches hold considerable promise at the experimental level, their transition toward large-scale commercialization and clinical adoption is constrained by stringent regulatory requirements. Chief among these is the need to rigorously establish clinical efficacy across heterogeneous populations, including variations in age, sex, and ethnicity. Addressing this demand entails large-scale clinical studies, which impose substantial temporal and economic burdens.

Although nucleic acid probe-based strategies have demonstrated great potential in the laboratory, their large-scale commercialization and clinical translation still face significant regulatory barriers. One key challenge is the requirement to demonstrate the clinical efficacy of the probes in the target population, ensuring reliable detection limits across patients of different ages, genders, and ethnic backgrounds. This necessitates large sample sizes and extensive clinical trials, which usually demand substantial time and financial resources. Furthermore, rigorous data must be provided to demonstrate the sensitivity and specificity of the strategy. What is the LOD of this method in patient cohorts known to carry the target point mutation compared to the gold standard, Can it reliably avoid false positives in healthy populations. In addition, it is essential to prove its ability to distinguish single-base differences without cross-reactivity, which requires testing with various potentially interfering mutant sequences. At the sample level, different types of specimens (blood, tissue, FFPE samples) may require distinct detection standards, necessitating additional validation data. Moreover, factors such as the type of collection tubes, storage temperature, and maximum storage duration must be standardized.

## 7 Integration of AI in nucleic acid probe-based mutation detection

Artificial intelligence (AI) has developed rapidly in recent years, covering fields such as medicine, engineering, and education, and has brought tremendous convenience to the world. In the medical domain, AI technology is widely used in disease diagnosis and precision medicine, demonstrating remarkable capabilities particularly in image recognition and genetic analysis. Although the integration of AI with nucleic acid probe-based detection is not yet mainstream, studies have shown that AI can effectively support such detection methods.

### 7.1 AI-assisted probe design

The design of nucleic acid probes involves potential secondary structures, which may lead to unexpected binding modes during hybridization with the target. Machine learning and AI technologies can predict these possible secondary structures. Combined with free energy analysis, they assist in designing more stable probes (Calonaci et al., 2020; Singh et al., 2019). Additionally, probe design must also consider factors such as complementarity to the target sequence and GC content. Deep learning-based AI approaches can further predict the binding efficiency between the probe and the target, facilitating the development of probes with higher specificity. Li et al. proposed a deep learning-based method named Deep DNAshape, which accurately accounts for the influence of extended flanking regions. It enables high-throughput prediction of DNA structural features for sequences of any length and quantity, thereby elucidating the impact of flanking regions on the structural properties of target DNA sequences (Li et al., 2024). This approach not only provides detailed structural analysis of probe-target binding but also offers valuable insights for the design of nucleic acid probes.

As early as 2009, studies had already embedded machine learning algorithms into thermodynamics-based primer design tools to accelerate the screening of efficient and specific primer pairs (Mann et al., 2009). In recent years, an increasing number of AI tools have been applied to the evaluation and optimization of primers and probes. For example, Dwivedi-Yu et al. introduced swga2.0, which combines active and machine learning to assess the amplification efficacy of individual primers and primer sets, enabling the design of primers for selective whole-genome amplification (Dwivedi-Yu et al., 2023). Similarly, Lin et al. developed a machine learning platform named BioInnovate AI. Compared to traditional tools such as Primer3 and Primer-BLAST, BioInnovate AI integrates key thermodynamic parameters to more accurately predict amplification success rates. By leveraging machine learning algorithms to predict the effectiveness of PCR primers and probes, the platform reduced PCR reagent development time by approximately 90%, significantly shortening the development timeline for diagnostic reagents targeting emerging infectious diseases (Lin et al., 2025).

### 7.2 AI-assisted signal analysis

The vast majority of nucleic acid probe-based detection methods rely on various forms of signal output, such as the fluorescence, electrochemical signals, and SERS signals mentioned above. However, when confronted with diverse and complex signals-such as background noise in fluorescence or intricate spectral profiles in SERS-manual interpretation or conventional algorithmic analysis tends to be time-consuming and susceptible to subjective bias. The development of AI models offers a promising new approach to signal interpretation. Through large-scale training, many AI-driven deep learning models can accurately decode complex signals, thereby improving both sensitivity and specificity (Zhou et al., 2024).

The optical filtering assembly, an essential component of fluorescence microscopy, helps suppress background noise while highlighting specific fluorescence signals. However, its use also increases the complexity of the fluorescence microscopy imaging system. To address this limitation, Dai et al. proposed a deep learning-based, filter-free fluorescence imaging method that leverages digital spectral filtering. This approach enables automatic selection of fluorescence channels and accurate prediction of fluorescence signals after image acquisition. Experimental results demonstrate that the method achieves excellent sensitivity and specificity across varying magnification levels (Dai et al., 2025). In addition, to address the problem of misjudgment caused by signal heterogeneity in conventional SERS detection, Zhao et al. developed an AI-driven SERS-LFIA automated detection system. This system innovatively integrates large-area SERS scanning imaging around the T-line with a deep learning model, enabling result interpretation based on the distribution patterns of nanoprobe signals rather than solely on signal intensity. The method achieved an accuracy of 94.52% in the test dataset (Zhao et al., 2025). Such examples of AI-based signal analysis pave the way for future advancements in nucleic acid probe signal interpretation.

### 7.3 Examples of AI-assisted mutation detection

With the rapid advancement of AI technology, an increasing number of models are being integrated into molecular diagnostics through deep learning applications in signal processing, pattern recognition, and predictive modeling. Although in most fields AI models still serve as auxiliary diagnostic tools, in several studies that have successfully incorporated AI into mutation detection, they play a significant role in assisting with result interpretation, signal analysis, and system optimization.

Sebuyoya and colleagues integrated an AI model into an electrochemical sensing platform employing RCA and recombinase polymerase amplification (RPA). This sensor is capable of detecting the *KRAS* G12V mutation at a frequency as low as 1% amidst a high background of wild-type sequences. Furthermore, when validated across seven cancer cell lines and 11 clinical samples from colorectal cancer patients, the results showed complete concordance with next-generation sequencing (NGS). The AI model enabled fully automated interpretation within this system and demonstrated the ability to perfectly distinguish complex cases-including samples with other mutations such as G12S and G13D intentionally introduced by researchers-a task that can be challenging for manual interpretation (Sebuyoya et al., 2025). Similarly, another research team employed a trained machine learning model to rapidly interpret results from CRISPR-Cas13-based lateral flow assays (LFA). As a widely used detection method, LFA offers advantages such as rapidity and low cost. However, visual interpretation by humans is prone to errors-especially when the test line is faint or under poor lighting conditions-potentially leading to false positives or false negatives. In contrast, the machine learning model can determine results within 0.2 s with an accuracy of 96.5%. More importantly, this high performance was consistently achieved across photos taken under diverse backgrounds (such as sofas, carpets, and beds), varying lighting conditions, and different smartphone angles and models (Xue et al., 2025).

Beyond conventional result interpretation, AI technology is also influencing other aspects of nucleic acid detection. In the AI-assisted droplet digital PCR (ddPCR) system developed by Colozza-Gama, a machine learning algorithm was employed to address a core challenge in ddPCR data analysis: automated droplet classification. Traditional methods often require manual classification by researchers, which introduces subjective variability between different operators. The optimized ddPCR platform ultimately achieved *BRAF* V600E detection comparable to the gold standard (pyrosequencing), and the correlation between automated classification results and the gold standard (R = 0.66) was significantly higher than that of manual classification (R = 0.55) (Colozza-Gama et al., 2021). This strategy not only offers a new approach to optimizing ddPCR, but also provides a foundation for integrating machine learning algorithms into ddPCR systems in the future. These advancements not only enhance the efficiency of nucleic acid probe-based detection in terms of time and economic cost but also elevate the overall standard of detection to a new level.

### 7.4 Challenges at the convergence of AI and laboratory

Although AI tools demonstrate significant potential in medical testing and disease diagnosis, their integration into laboratory settings and eventual application in clinical diagnostics face considerable challenges. The development of every AI model requires a large volume of high-quality samples. However, differences in instruments and environmental conditions across laboratories may lead to data inconsistencies. A well-trained model may exhibit suboptimal outcomes when applied in other locations. Additionally, many complex AI models suffer from the “black box” problem-lacking interpretable biological explanations-which hinders their adoption in clinical practice. This represents one of the major obstacles that current AI models must overcome. Moreover, for a mature AI tool to be deployed in clinical settings, it must undergo rigorous clinical trials to demonstrate its efficacy and robustness, and obtain approval from regulatory agencies such as the FDA in the United States. This is a time-consuming process. Given the rapid pace of AI development, a more advanced model may emerge before its predecessor even completes regulatory review. Finally, the development of AI systems demands substantial expertise in computer science and often requires a team of specialized professionals. For medical researchers and conventional laboratories, this presents a significant barrier to entry.

## 8 Summary and outlook

Mutations are closely associated with disease susceptibility, pathogenesis, and therapeutic outcomes. The detection of relevant point mutations is crucial for early disease prevention and diagnosis. In recent years, DNA point mutation detection has undergone rapid development, evolving from traditional methods such as Sanger sequencing and PCR to more advanced approaches like NGS and TGS, all of which have achieved remarkable success. However, these methods are often limited either by low throughput and suboptimal performance in identifying low-frequency mutations, or by high costs and complex data analysis. In the current global healthcare context, there is a growing demand for rapid and accurate screening of diseases at early stages. Therefore, there is an urgent need to develop detection technologies with higher sensitivity, enhanced specificity, and simplified signal readouts. This review summarizes the design strategies for point mutation detection using nucleic acid probes, with a focus on the applications of enzymatic reactions, nanotechnology, nucleic acid derivatives, and pure nucleic acid probes. Additionally, we discuss emerging AI-assisted detection technologies at the end of the article, The advantages and disadvantages of each approach are summarized in Table 3. A comparative analysis of each specific method is provided in Table 4.

**TABLE 3:** Advantages and disadvantages of various point mutation detection techniques.

Based point mutations detection	Advantages	Disadvantages
Sanger	Technologically mature, High accuracy	Low sensitivity, High cost
NGS	High-throughput, High sensitivity, Well-suited for complex mutations	Complex data analysis, High error rate, Requires relatively specialized equipment
TGS	Long-reads, More suitable for detecting large-scale variations	High cost, Low adoption rate, Time-consuming
Guided enzymes	Technological maturity, Programmability, Low cost, Broad application potential	Limitations of PAM sequences, Off-target effects, Delivery challenges, Immune response
Ligase	Low sample requirement, Multiplex detection capability, High sensitivity	Thermal instability, Low catalytic efficiency, Complex probe design
DNAzyme	Low cost, Programmability, High stability, Low immunogenicity	Low catalytic efficiency, Metal ion-dependent, Complex synthesis
Nanozyme	High stability, Versatile design, Easy to synthesize, High sensitivity	Potential toxicity, Challenges in large-scale production, Low specificity
Enzyme-free	Low cost, Programmability, Easy to operate	High design complexity, Low sensitivity, Restricted *in vivo* application
Colorimetry	Simple and rapid operation, Visual readout, High sensitivity	Background interference, Sensitive to reaction conditions, Difficult to quantify
Fluorescence methods	High sensitivity, Quantitative analysis capability, Multiplex detection	Background interference, High cost, Requires sophisticated equipment
Electrochemical methods	High sensitivity and specificity, Rapid detection	Complex operational procedure, Limited clinical applicability
SERS	Multiplex detection, High sensitivity and specificity, Low sample consumption, Rich molecular information	Requires sophisticated equipment, Difficult to quantitative detection, Complex signal analysis, Requires sophisticated probe design
PNA	High affinity, Resistant to enzymatic degradation, High specificity	Poor aqueous solubility, Inefficient cellular internalization, High production costs
LNA	Enhanced binding affinity, High stability, Compatibility with various detection platforms	Probe structural rigidity, Potential toxicity risks, Complex probe design
Pure nucleic acid probe	Programmable structure and function, Multiplatform compatibility	Insufficient dynamic regulation, Limited interference resistance
AI-assisted	High accuracy, High efficiency, Minimal errors and biases	Technologically immature, Black box effect, High technical requirements

**TABLE 4:** Comparison of techniques for detecting gene mutations.

Method	Target	Applications	Sensitivity	References
Enzymatic approaches				
CRISPR-Cas9-assisted REs	*UME6*	Candidiasis	4%	Evans et al. (2018)
SgRNA design	*EGFR*	NSCLC	0.1%	Bae et al. (2019)
DASH	*KRAS* G12D, rRNA	Multiple cancers, Meningitis	0.1%	Gu et al. (2016)
Cas9-AFLPA	*EGFR*, *HBB*	Somatic mutations	1%–10%	Jia et al. (2018)
PAND	*BRCA1*, *EGFR*	Multiple cancers	10^–19^ mol/L	He et al. (2019)
Ago-GFET	miRNA, cfDNA, viral RNA	SARS-CoV-2 viral infection, Influenza virus infection	10^–20^ mol/L	Kong et al. (2024)
NAVIGATER	*KRAS*, *EGFR*	Pancreatic cancer	0.01%	Song et al. (2020)
PCR-LDR-qPCR	*BRAF*, *TP53*	Multiple cancers	0.02%	Ruiz et al. (2020)
eLCR	*CYP2C19*	Drug metabolism	0.5 × 10^−15^ mol/L	Liu et al. (2020b)
LDR-assisted fluorescence assay	*KRAS* G12V	Colorectal cancer, Pancreatic cancer	5%	Sawamura and Hashimoto (2017)
Ligation-bHCR	*KRAS* G12V	Colorectal cancer	1%	Tang et al. (2017)
Ligation-RCA	*HBB*	β-thalassemia	40 × 10^−15^ mol/L	Zou et al. (2014)
Cas14a-LCR-RCA	*BRAF* V600E	Papillary thyroid carcinoma	0.3 × 10^−16^ mol/L	Shi et al. (2022)
AuNP-assisted RCA	β-thalassemia gene	Thalassemia	7 × 10^−14^ mol/L	Li et al. (2010)
DNAzymes-LCR	β-globin gene	sickle cell anemia	20 copies/μL	Zhou et al. (2013)
DNAzymes-CHA-CL	*MAPT*	Alzheimer’s disease	3 × 10^−16^ mol/L	Xu et al. (2017)
DNAzymes-FEN1-CHA	*KRAS*	Colorectal cancer, Pancreatic cancer	4.23 × 10^−15^ mol/L	Li et al. (2022)
H-GN-probe-TMB-H_2_O_2_	HBV DNA	Hepatitis B	2 × 10^−9^ mol/L	Guo et al. (2011)
MS-PtNPs-probe- assisted colorimetry	*M. tuberculosis* DNA	Tuberculosis	2.6 × 10^−9^ mol/L	Chen et al. (2018)
Nanomaterial approaches				
AuNPs-assisted colorimetry	*KRAS*	Colorectal adenocarcinoma	100 × 10^−7^ mol/L	Sato (2005)
Label-free-AuNPs-assisted colorimetry	*EGFR*	NSCLC	313 × 10^−9^ mol/L	Ramanathan et al. (2019)
TC-AuNPs-assisted colorimetry	Pathogen DNA	*Staphylococcus aureus*	2 × 10^−14^ mol/L	Ganguly et al. (2021)
AuNPs-assisted IR-NEANA	*EGFR*	Cancer somatic cell screening	10^–12^ mol/L	Zou et al. (2015)
QD-FRET DNA nanosensor	*KRAS*	Ovarian serous borderline tumor	<50 copies/μL	Zhang et al. (2005a)
QD-assisted dual-color fluorescence assay	β-globin gene, *KRAS*	Ovarian Serous Borderline Tumors	10^–21^ mol/L	Yeh (2006)
CdTe-assisted fluorescence assay	*EGFR*	NSCLC	2%	Kang et al. (2012)
UCNPs-LRET fluorescence assay	*HBB*	Sickle Cell Disease	6 × 10^−10^ mol/L	Kumar et al. (2009)
NIR probe-assisted fluorescence assay	*EGFR* T790M	NSCLC	5 × 10^−12^ mol/L	Wang et al. (2014b)
SLR-AuNP electrochemical sensor	*KRAS* G12D	Multiple cancers	10^–12^ mol/L	Wang et al. (2011b)
MWCNT-AuNP electrochemical sensor	*TP53*	Multiple cancers	10^–17^ mol/L	Fayazfar et al. (2014)
GO-GCE electrochemical sensor	DNA	Human serum sample	1 × 10^−17^ mol/L	Huang et al. (2015)
CdTe QDs	PC3 cell	Prostate cancer, *Escherichia coli*	5 × 10^−10^ mol/L	Moulick et al. (2017)
QD electrochemical sensor	*ApoB100* R3500Q	Hypercholesterolemia, Atherosclerosis	3.4 × 10^−17^ mol/L	Mazloum-Ardakani et al. (2015)
CdS QD-ECL sensor	*EGFR* T790M	NSCLC, Lung Cancer	3.4 × 10^−18^ mol/L	Yang et al. (2023)
AuNPs-mLAMP	*CYP2C19*	Cervical cancer	100 copies/μL	Lu et al. (2017)
rGO-AuNP-ePCR	*EGFR*	Lung cancer	5 copies/μL	Wang et al. (2022)
PCR-SERS	*BRAF* V600E, *PIK3CA* E542K	Colorectal cancer	4%–6%	Li et al. (2018)
Asy-PCR-SERS	*KRAS* G12V	Colorectal cancer, Lung Cancer	0.1%	Lyu et al. (2021)
Au@Ag NRs	*KRAS*	Multiple cancers	5 × 10^−14^ mol/L	Wu et al. (2019)
AgNPs@Si SERS	*SMAC/DIABLO* C377T	Deafness	5 × 10^−13^ mol/L	Wang et al. (2014a)
SNA-GTNA SERS	HBV DNA	Hepatitis B	5 × 10^−17^ mol/L	Li et al. (2013)
Nucleic acid analogs approaches				
PNA-PCR	*EGFR*	NSCLC	N/A	Kim et al. (2013)
PNA-PCR-FISH	*BCR-ABL* T315I	CML	0.5%	Rosso et al. (2015)
PNA-Pdx	SARS-CoV-2 genome	COVID-19	2 copies/μL	Jiang et al. (2023)
LNA-PCR	*FLT3*, *JAK2*	AML, MPN	1%	Warshawsky and Mularo (2011)
LNA-PNA-PCR	*KRAS* G12D,*KRAS* G12V	Pancreatic Cancer	0.1%	Itonaga et al. (2016)
LNA-AuNPs detection	hemolysin-A	Foodborne illness	4 × 10^−10^ mol/L	Rastogi et al. (2012)
LNA-MNPs-SERS	hsa-miR-200a-3p	Ovarian cancer	2 × 10^−16^ mol/L	Wang et al. (2024b)
LNA-involved biosensor	*EGFR* L858R, *KRAS* G12D	NSCLC	1%	Tian et al. (2018)
Pure nucleic acid probe approach				
TSCP	*PIK3CA* E545K	Breast cancer	10^–9^ mol/L	Wu et al. (2025)
SELECT	*KRAS* G12D, G13D	Ovarian cancer	0.1%	Chen et al. (2019)
Fluorescent probe- TIRFM	*KRAS*	Lung cancer	0.01%	Su et al. (2017)
CHA-FRET-TMSD	*KRAS*	Multiple cancers	4.3 × 10^−15^ mol/L	Zhang et al. (2024)
AI-assisted detection method				
AI-RCA-RPA	*KRAS* G12V	Colorectal cancer	1%	Sebuyoya et al. (2025)
AI-Cas13-LFA	SARS-CoV-2 gene	SARS-CoV-2	10^−9^ mol/L	Xue et al. (2025)
AI-ddPCR	*BRAF* V600E	Papillary Thyroid Carcinoma	10%	Colozza-Gama et al. (2021)

As sequencing technologies continue to mature, it is expected that numerous detection strategies compatible with TGS will be developed. Furthermore, with the rapid advancement of AI, many novel probe design concepts and analytical strategies are likely to emerge in the field of mutation detection. Additionally, the establishment of large-scale datasets and continuous deep learning will drive the development of more comprehensive and efficient models. These advances may help address unresolved biological problems and unpredictable kinetic processes. In the future, AI models specifically tailored for mutation detection-rather than merely serving auxiliary roles-could be developed and even integrated with clinical diagnostics, thereby driving progress in precision medicine.
